# Mitofusin 2 sustains the axonal mitochondrial network to support presynaptic Ca^2+^ homeostasis and the synaptic vesicle cycle in rat hippocampal axons

**DOI:** 10.1523/JNEUROSCI.1356-22.2023

**Published:** 2023-03-30

**Authors:** Jason D. Vevea, Edwin R. Chapman

**Affiliations:** 1 Department of Neuroscience, University of Wisconsin-Madison, 1111 Highland Ave., Madison, WI, 53705; 2 Howard Hughes Medical Institute, 1111 Highland Ave., Madison, WI, 53705; 3 Current address: Department of Developmental Neurobiology, St. Jude Children’s Research Hospital, 262 Danny Thomas Pl., Memphis, TN, 38105

## Abstract

Mitochondria exert powerful control over cellular physiology, contributing to ion homeostasis, energy production, and metabolite biosynthesis. The trafficking and function of these organelles are particularly important in neurons, with impaired mitochondrial function or altered morphology observed in every neurodegenerative disorder studied. While mitochondrial biosynthetic products play a crucial role in maintaining cellular function, their resulting byproducts can have negative consequences. Thus, organelle quality control (QC) mechanisms that maintain mitochondrial function are imperative to restrict destructive signaling cascades in the cell. Axons are particularly sensitive to damage, and there is little consensus regarding the mechanisms that mediate mitochondrial QC in this compartment.

Here, we first investigated the unstressed behavior of mitochondria in rat hippocampal neurons of mixed sex, focusing on mitochondrial trafficking and fusion to better understand potential QC mechanisms. We observed size and redox asymmetry of mitochondrial traffic in axons, suggesting an active QC mechanism in this compartment. We also document biochemical complementation upon the fusion and fission of axonal mitochondria. Eliminating fusion by knocking down the neuronal mitochondrial fusion protein mitofusin 2 (MFN2) reduced the rates of axonal mitochondrial trafficking and fusion, decreased the levels of synaptic vesicle (SV) proteins, inhibited exocytosis, and impaired SV recruitment from the reserve pool during extended stimulation. MFN2 knockdown also resulted in presynaptic Ca^2+^ dyshomeostasis. Remarkably, upon MFN2 knockdown, presynaptic mitochondria sequestered Ca^2+^ more efficiently, effectively limiting presynaptic Ca^2+^ transients during stimulation. These results support an active mitochondrial trafficking and fusion-related QC process that supports presynaptic Ca^2+^ handling and the SV cycle.

## Introduction

Mitochondria play important roles in numerous cellular functions by supporting energy production (adenosine triphosphate, ATP), metabolite biosynthesis (lipids, amino acids, nucleotides, heme), ion homeostasis (K^+^, Ca^2+^), and the response to cellular stress (Spinelli and Haigis, 2018). Mitochondria exhibit dynamic changes in their structure and appearance, and these differences in shape and form between different types of cells may indicate that they serve specific functions unique to those cells. Indeed, these various mitochondrial morphologies arise from differences in motor activity, expression of organelle anchors, and the balance of mitochondrial fusion to fission (Eisner et al., 2018). Mitochondrial fusion also serves to increase mitochondrial network homogeneity (Youle and van der Bliek, 2012). Specifically, fusion of new, higher-functioning mitochondria with aged or damaged peripheral mitochondria can result in complementation, rejuvenating the less-fit population of these organelles (Chen et al., 2005).

Mitochondrial morphology can differ dramatically even within a single cell. For example, neurons broadly consist of three compartments: a soma or cell body that houses the nucleus and an interconnected mitochondrial network; dendrites that are specialized to receive chemical signals and have parallel arrays of interconnected mitochondria; and an axon that maintains synaptic vesicles through the synaptic vesicle (SV) cycle, with shorter, well-separated mitochondrial units (Lewis et al., 2018; Faitg et al., 2021). Notably, mitochondrial size and connectedness directly correlate with the organelles’ Ca^2+^ buffering capacity (Lewis et al., 2018). So, mitochondria in the axon likely have a lower overall capacity to buffer Ca^2+^ relative to other neuronal compartments, and the Ca^2+^ buffering capability of the shorter and less contiguous axonal network of mitochondria may be particularly sensitive to perturbation, resulting in the partial disruption of axonal function (e.g., the SV cycle). Indeed, presynaptic Ca^2+^ buffering by mitochondria can influence presynaptic Ca^2+^, and thus, neurotransmitter release and the SV cycle (Tang and Zucker, 1997; Billups and Forsythe, 2002; Kwon et al., 2016; Vaccaro et al., 2017). The SV cycle is regulated by Ca^2+^ at many stages. Broadly, the cycle is composed of SV docking, priming, and fusion with the presynaptic membrane, thus releasing neurotransmitters into the synaptic cleft. SV constituents are reinternalized, followed by sorting, and finally SV reformation (Chanaday et al., 2019). How mitochondrial quality control (QC) pathways support mitochondrial Ca^2+^ buffering capacity in the axon and which steps of the SV cycle are influenced when these pathways are disrupted remain open questions. Understanding these pathways will better inform the mechanistic study of neurodegenerative disorders that show signs of altered mitochondrial morphology or altered presynaptic Ca^2+^ homeostasis.

Here, we first examined axonal mitochondrial morphology, trafficking, and redox status in unstressed neurons by using fluorescent probes and microfluidic devices. We observed mitochondrial trafficking asymmetry with respect to size and redox status, indicating an axonal QC mechanism. We also provide evidence for redox complementation during mitochondrial fusion and content-mixing events in axons. We then evaluated whether axonal mitochondrial fusion is critical for the function of the synaptic vesicle (SV) cycle by knocking down the main neuronal mitochondrial outer membrane fusion protein, mitofusin 2 (MFN2) (Eura et al., 2003). We utilized an SV-targeted pHluorin (vGlut1-pHluorin) (Voglmaier et al., 2006) to monitor the SV cycle and observed decreased exocytosis and a reduction in SV pool mobilization in MFN2 knock down (KD) neurons. We next examined presynaptic Ca^2+^ homeostasis using: a) SV-targeted GCaMP6f (SYP-GCaMP6f), b) a fast far-red Ca^2+^ sensor HTL-JF646-BAPTA-AM (Deo et al., 2019) targeted to SVs by using synaptophysin-HaloTag (SYP-HT) (Bradberry and Chapman, 2022), and c) a mitochondrial matrix–targeted Ca^2+^ sensor (mito-GCaMP5G) (Kwon et al., 2016). In MFN2 KD neurons, there was no change in Ca^2+^ influx; however, there was faster clearance of presynaptic [Ca^2+^]_i_ that is mediated by an enhanced ability of mitochondria to sequester Ca^2+^. Together, these optical biosensors revealed that super-physiological mitochondrial Ca^2+^ uptake, originating from MFN2 disruption, may drive axonal Ca^2+^ alterations to decrease the SV pool, inhibit vesicle pool mobilization, and sensitize mitochondria to Ca^2+^ overload.

## Materials and Methods

### Ethics Statement

Animal care and use in this study were conducted under guidelines set by the NIH Guide for the Care and Use of Laboratory Animals handbook. Protocols were reviewed and approved by the Animal Care and Use Committee (ACUC) at the University of Wisconsin, Madison (Laboratory Animal Welfare Public Health Service Assurance Number: A3368-01).

### Cell Culture

Rat (Sprague Dawley) hippocampal and cortical neurons were isolated from E18 pups of both sexes (Envigo) by using a procedure previously described in (Vevea and Chapman, 2020). In brief, rat hippocampal neurons were dissected, treated with trypsin (Corning; 25-053-CI), triturated, and plated on glass coverslips (Warner instruments; 64-0734 (CS-18R17)) previously coated with poly-D-lysine (Thermo Fisher Scientific; ICN10269491). Hippocampal neurons were also cultured in standard neuron microfluidic devices (SND450, XONA Microfluidic devices) mounted on glass coverslips, as previously described (Bomba-Warczak et al., 2016). Neuronal cultures were grown in Neurobasal-A (Thermo Fisher; 10888-022) medium supplemented with B-27 (2%, Thermo Fisher Scientific; 17504001), GlutaMAX (2 mM Gibco; 35050061) and penicillin/streptomycin solution (1%, Thermo Fisher Scientific; MT30001CI) before experiments. All experiments were performed up to 20 days in vitro (DIVs); 13-16 DIV for microfluidic experiments and 14-20 DIV for pHluorin, Ca^2+^ (GCaMP#X/JF646-BAPTA-AM), roGFP, and immunoblot experiments. For lentivirus preparation, HEK293T cells (ATCC) were cultured following ATCC guidelines. These cells had been previously tested for mycoplasma contamination by using the Universal Mycoplasma Detection Kit (ATCC; 30-1012K). The HEK293T cells were also validated via short tandem repeat profiling by ATCC (ATCC; 135-XV).

### Lentivirus production and use

Lentivirus production was performed as described previously (Vevea and Chapman, 2020). When needed, lentiviral constructs were subcloned into the FUGW transfer plasmid (FUGW was a gift from David Baltimore (Addgene plasmid # 14883 ; http://n2t.net/addgene:14883 ; RRID:Addgene_14883)) (Lois et al., 2002). We had previously replaced the ubiquitin promoter with the CAMKII promoter or human synapsin I promoter (Kugler et al., 2003; Vevea and Chapman, 2020). Lentivirus was added to neuronal cultures between 2 DIV and 5 DIV.

### Plasmid use and construction

The redox-sensitive green fluorescent protein (pEGFP-N1-mt-ro1GFP) was a gift from S. James Remington (Addgene plasmid # 82407 ; http://n2t.net/addgene:82407 ; RRID:Addgene_82407). The mitochondrial targeted Ca^2+^ sensor pCAG mito-GCaMP5G was a gift from Franck Polleux (Addgene plasmid # 105009 ; http://n2t.net/addgene:105009 ; RRID:Addgene_105009) (Kwon et al., 2016). Silencing constructs encoding control shRNA and *mfn2* shRNA (target sequence: TGGATGGACTATGCTAGTGAA) were purchased from Sigma (SHC202 and TRCN0000080612, respectively). The vGLUT1-pHluorin construct (Voglmaier et al., 2006) was subcloned into our previously modified lentivirus backbone of choice derived from FUGW (Vevea and Chapman, 2020). For measuring synaptic Ca^2+^, the HaloTag cassette from pHTC HaloTag CMV-neo Vector (Promega; G7711) was PCR-amplified and appended to the carboxy-terminus of synaptophysin (SYP) with a GS(GSS)_4_ linker and subcloned into the modified FUGW lentiviral vector. Presynapse-targeted GCaMP6f (SYP-GCaMP6f) was described previously (Bradberry and Chapman, 2022). Mitochondrial matrix–targeted GFP, cytosolic mCherry, and photoactivatable GFP were subcloned into pEF-GFP after excising the GFP (pEF-GFP was a gift from Connie Cepko (Addgene plasmid # 11154 ; http://n2t.net/addgene:11154 ; RRID:Addgene_11154)) (Matsuda and Cepko, 2004). Note that the fluorescent proteins GFP, PA-GFP, and mCherry used here lack the carboxy terminal MDELYK sequence, as this sequence was observed to promote protein aggregation in long-lived cells (unpublished observation, Dr. Joseph S. Briguglio).

### Live-cell image acquisition

**Figure 1:**
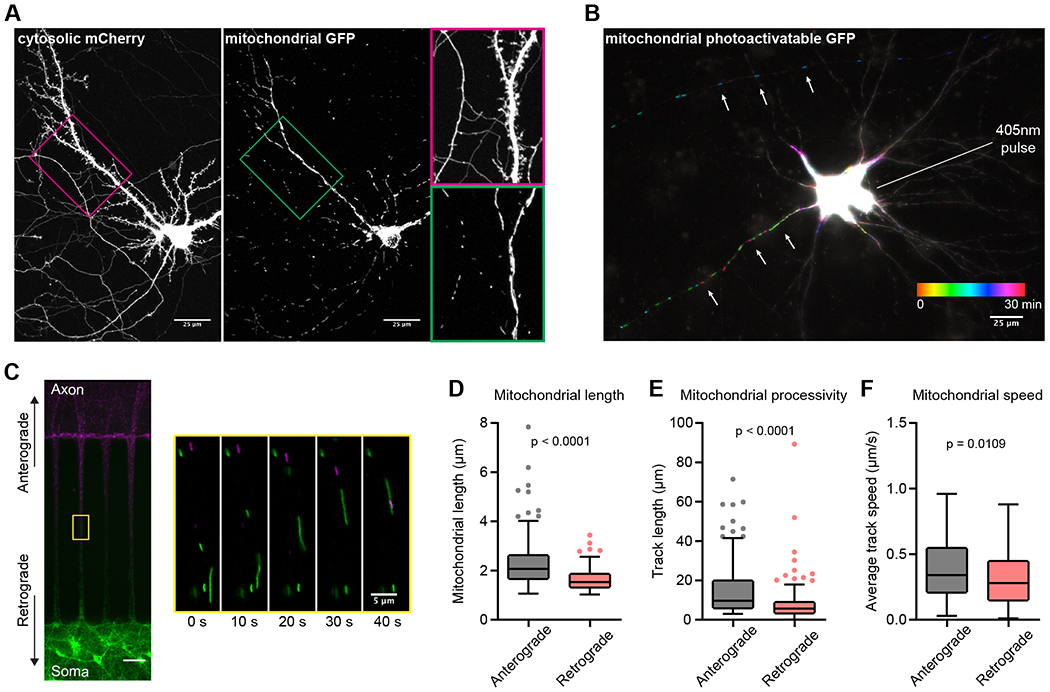
Asymmetric trafficking of mitochondria in axons **A**) Maximum projection of a live-cell, confocal z-stack from a 16 DIV neuron co-transfected with cytosolic mCherry and mitochondrial matrix–targeted, monomeric-superfolder green fluorescent protein (msGFP). Scale bar: 25 μm. Magenta and green boxes outline zoomed-in areas on the right, highlighting the distinct morphologies of axonal and dendritic mitochondria. **B**) Temporal color-coded maximum projection of a 15 DIV neuron expressing mitochondrial matrix–targeted, photo-activatable GFP (PA-GFP). Mitochondrial soma egress was monitored after photoactivation by imaging every minute for 30 total minutes. Scale bar: 25 μm. **C**) Representative images of 14 DIV neurons, grown in SND450 (Xona) microfluidic devices, stained with MitoTracker Green FM (MTG) and MitoTracker Red CMXRos (MTR) on the soma and axon sides, respectively. Scale bar: 50 μm. The yellow square marks the area enlarged on the right, showing delineated mitochondrial trafficking events at the indicated time points. Scale bar: 5 μm. Mitochondria imaged in this area have traveled at least 200 μm, as the microchannels in the microfluidic device are 450-μm long. **D**) Mitochondria were segregated into anterograde (black) or retrograde (red) groups based on MitoTracker dye labeling (green vs. red) and direction of motility in the microchannel (see also Materials and Methods). Mitochondrial lengths are quantified from the middle of the microchannel: anterograde = 2.07 μm [1.88 – 2.15] and retrograde = 1.53 μm [1.46 – 1.62]. Values are medians with 95% confidence interval (CI) representing error, Mann-Whitney test U = 6152, n_1_ = 165, n_2_ = 160, p < 0.0001. **E**) Track lengths (processive movement or un-paused motility); anterograde (black) = 9.64 μm [7.82 – 12.35] and retrograde (red) = 5.67 μm [4.64 – 6.06], segregated as in panel D. Pauses were defined as three consecutive images (15 s each) where the organelle did not move. Values are medians with 95% CI representing error, Mann-Whitney test U = 7401, n_1_ = 165, n_2_ = 160, p < 0.0001. **F**) Average track speed values (track distance over time) plotted by anterograde (black) or retrograde (red) direction, segregated as in panel D; anterograde (0.34 μm/s [0.30 – 0.39]) and retrograde (0.28 μm/s [0.24 – 0.31]) mitochondrial trafficking speeds are plotted. Values are medians with 95% CI representing error, Mann-Whitney test U = 11044, n_1_ = 165, n_2_ = 160, p = 0.0109. Graphs in panels D-F are Tukey box and whisker plots. Data were gathered from at least three independent experiments; each n represents an individual anterograde- or retrograde-trafficking mitochondrion.

**Figure 2:**
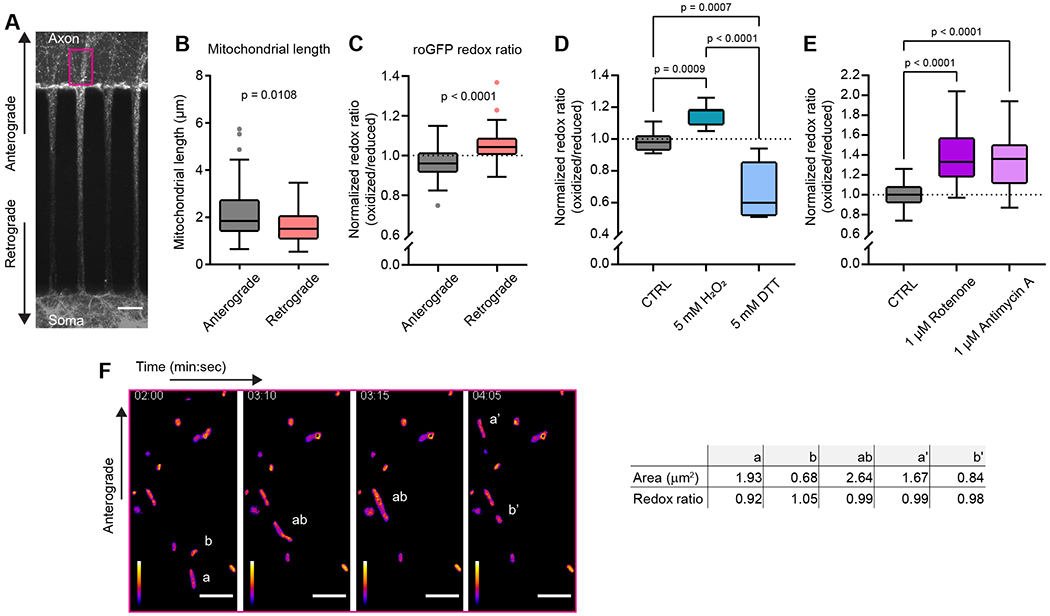
Anterogradely trafficked mitochondria are more reduced and complement resident axonal mitochondria **A**) Representative images of neurons grown in SND450 (Xona) microfluidic devices and transfected with mitochondrial matrix–targeted redox-sensitive GFP (roGFP). Analysis in 2B-C and 2F was limited to fields of view (FOVs) on the axon side near microchannels, denoted by the magenta rectangle. Scale bar = 50 μm. **B**) As in Figure 1D, we compared the length of anterograde-trafficked mitochondria (black) to that of retrograde-trafficked mitochondria (red). Anterograde-trafficked mitochondria were again longer than retrograde-trafficked mitochondria, with lengths for anterograde mitochondria of 1.84 μm [1.63 – 2.27] and retrograde mitochondria, 1.52 μm [1.19 – 1.95]. Values are medians with 95% CI representing error, Mann-Whitney test U = 1024, n_1_ = 54, n_2_ = 53, p = 0.0108. Charts in panels B and C are Tukey box and whisker plots with data from at least three independent experiments; each n represents an individual anterograde- or retrograde-trafficking mitochondrion. **C**) The redox ratio was calculated for anterograde- (black) or retrograde- (red) trafficking mitochondria as described in Materials and Methods, then normalized to the average redox ratio of the population of mitochondria in the FOV. The redox ratio values were 0.96 [0.94 – 0.97] (oxidized to reduced) for anterograde and 1.04 [1.02 – 1.06] (oxidized to reduced) for retrograde. Values are medians with 95% CI representing error, Mann-Whitney test U = 454, n_1_ = 54, n_2_ = 53, p < 0.0001. **D**) Quantitation of mito-roGFP dynamic range after 30-minute exposure to control (PBS), oxidizing (5 mM H_2_O_2_), or reducing (5 mM Dithiothreitol, DTT) treatment. Redox ratios are from paired measurements and normalized to control (CTRL) values. The redox ratios were 0.98 [0.96 – 1.01] (oxidized to reduced) for CTRL (black), 1.18 [1.13 – 1.18] (oxidized to reduced) for H_2_O_2_ (teal), and 0.60 [0.59 – 0.74] (oxidized to reduced) for DTT (azure). Values are medians with 95% CI representing error, Kruskal–Wallis test H = 53.21, n_1_ = 21, n_2_ = 21, n_3_ = 21, p < 0.0001. Follow-up of multiple comparisons by applying Dunn’s correction resulted in the following p-values: CTRL vs. H_2_O_2,_ p = 0.0009; CTRL vs. DTT, p = 0.0007; and H_2_O_2_ vs. DTT, p < 0.0001. Charts in panels D and E are Tukey box and whisker plots from three independent experiments; each n represents the soma and dendritic arbor of an individual neuron from a mass-dissociated culture after sparse transduction with mito-roGFP lentivirus. **E**) Quantitation of the mito-roGFP dynamic range after 24 hours of complete inhibition of electron transport chain by the complex I inhibitor rotenone (1 μM ROT) and the complex III inhibitor antimycin A (1 μM AA). Redox ratios are normalized to control (DMSO/vehicle, CTRL) values at the 24-hour timepoint. The redox ratios were 1.00 [0.95 – 1.05] (oxidized to reduced) for CTRL (black), 1.33 [1.29 – 1.51] (oxidized to reduced) for rotenone (violet), and 1.36 [1.26 – 1.43] (oxidized to reduced) for antimycin A (lilac). Values are medians with 95% CI representing error, Kruskal–Wallis test H = 38.96, n_1_ = 28, n_2_ = 31, n_3_ = 37, p < 0.0001. Follow-up multiple comparisons using Dunn’s correction resulted in the following p-values: CTRL vs. ROT, p < 0.0001; CTRL vs. AA, p < 0.0001. **F**) Representative images from an image series of axonal mito-roGFP. Low redox ratio mitochondria (*a*) can be seen moving in the anterograde direction toward stationary mitochondria (*b*). These mitochondria fuse and the redox ratio equilibrates as the now single mitochondrion (*ab*) continues in the anterograde direction. After a short distance, it divides and a small piece, approximately the size of the original (*b*) mitochondrion, is left in a new position (*b’*). The rest of the mitochondrion (*a’*) continues in the anterograde direction. A table of size and redox ratio values is listed beside the images. White scale bar: 5 μm, Fire LUT scale bar represents intensity: white = highest ratio, purple = lowest ratio.

**Figure 3:**
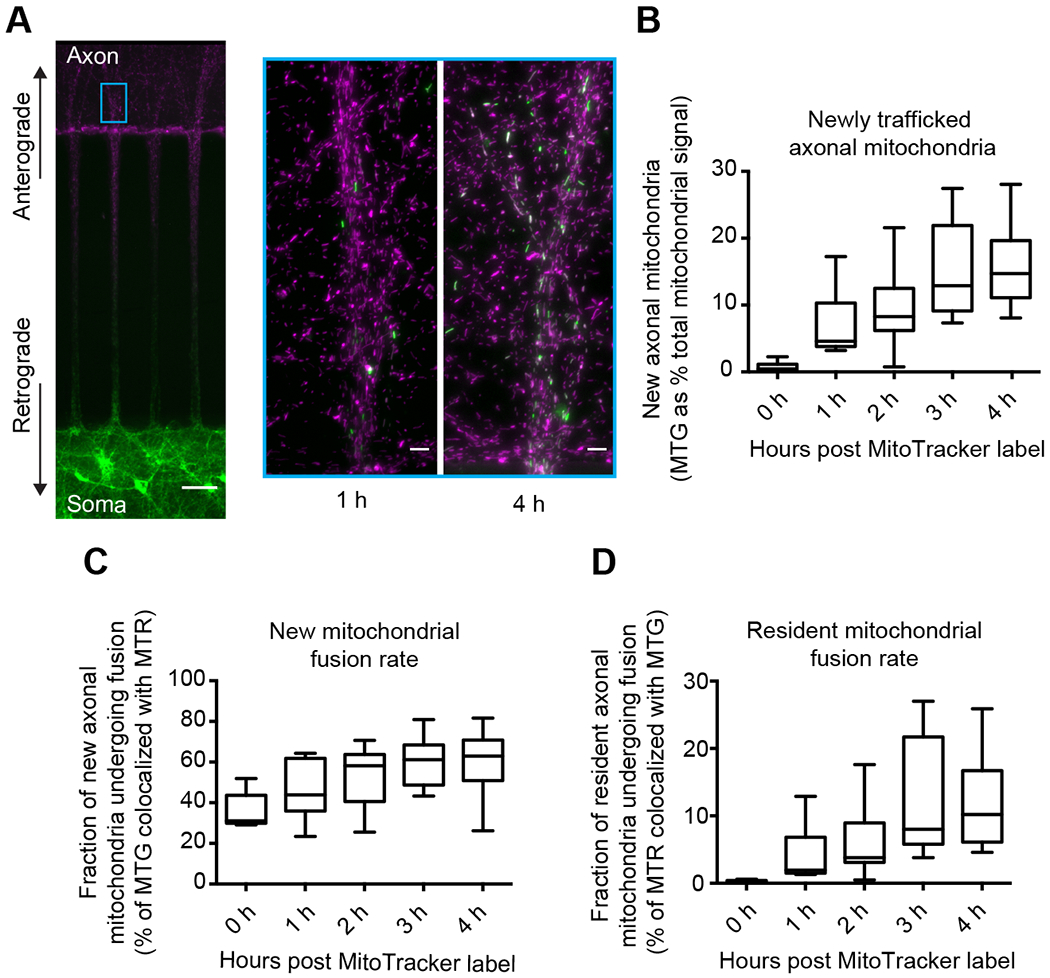
Robust anterograde axonal mitochondrial trafficking complements the resident axonal mitochondrial network **A**) Representative image (left) of neurons grown (13-16 DIV) in a microfluidic device as in Fig. 1C. Analysis of data in panels B-D was limited to fields of view (FOVs) on the axon side near microchannels, denoted with the cyan rectangle. Scale bar is 50 μm (left) and 5 μm for enlarged areas. The enlarged area bound by the cyan square shows representative images of 1 hour and 4 hours post MitoTracker labeling, displaying somato-dendritic mitochondrial traffic into axons (MTG) and fusion with resident axonal mitochondria (MTR). **B**) A measure of newly trafficked axonal mitochondria, defined by MTG signal as a percentage of total fluorescent signal (MTG/(MTR + MTG)) at hour intervals post MitoTracker labeling. Newly trafficked mitochondria account for 4.59% [3.8% - 10.72%] of total mitochondria at 1 hour, 8.28% [6.25% - 12.4%] at 2 hours, 12.9% [9.13% - 21.91%] at 3 hours, and 14.71% [11.19% - 19.32%] at 4 hours. Values are medians with 95% CI representing error. **C**) A measure of the fraction of new mitochondria (visualized with MTG) that fuse with resident axonal mitochondria, defined as the percentage of MTG signal that colocalized with MTR signal. The fraction of newly trafficked mitochondria that undergo fusion is 43.9% [35.8% - 61.9%] of new mitochondria at 1 hour, 58.2% [42.9% - 63.4%] at 2 hours, 61.2% [48.7% - 68.4%] at 3 hours, and 62.9% [56.6% - 68.7%] at 4 hours. Values are medians with 95% CI representing error. **D**) Fraction of resident axonal mitochondria (visualized with MTR) that fuse with newly trafficked mitochondria, defined as the percentage of MTR that colocalized with MTG. These values are: 1.95% [1.5% - 7.7%] of new mitochondria at 1 hour, 3.8% [3.1% - 8.2%] at 2 hours, 8% [5.8% – 21.7%] at 3 hours, and 10.2% [6.4% - 15.7%] at 4 hours. Values are medians with 95% CI representing error. Charts in panels B-D are Tukey box and whisker plots with n = 5 for time 0 hour and n > 15 for each other group; data are from at least three independent experiments. Each n represents an FOV from the axon chamber.

**Figure 4:**
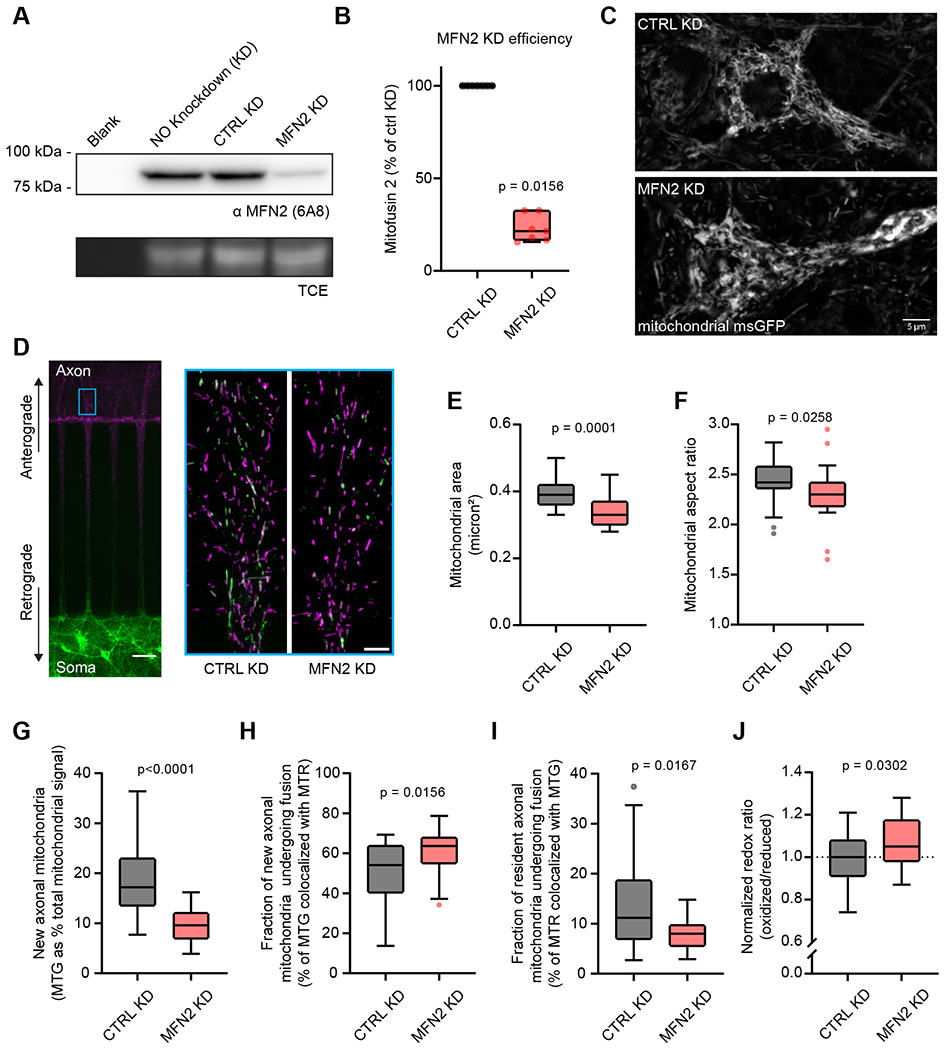
Mitofusin 2 supports mitochondria morphology, anterograde trafficking, and mitochondrial complementation in axons **A)** Representative anti-MFN2 immunoblot from rat 15 DIV hippocampal neurons with trichloroethanol (TCE) as a loading control. Conditions from left to right: blank, no protein; control, no lentivirus application; control, non-targeting shRNA lentivirus (CTRL KD); experiment, treated with MFN2-targeted shRNA (see Materials and Methods) (MFN2 KD). **B**) A Tukey box and whisker plot of MFN2 knockdown efficiency quantitated by immunoblot densitometry from 7 independent dissociated neuronal samples. Median MFN KD (red) percentage is 21.45% [95% CI: 15.6% - 32.7%], Wilcoxon signed-rank test, p = 0.0156. **C**) Representative Airyscan images of single focal planes through the soma and proximal dendrites of neurons transduced with mitochondrial-matrix–targeted msGFP, and CTRL or MFN2 KD lentivirus constructs. Mitochondrial aggregation and loss of tubular network, characteristic of loss of MFN2 function, is visible in the MFN2 KD–treated neurons. Scale bar: 5 μm. **D**) Representative image (left) of neurons grown (13-16 DIV) in a microfluidic device as in Fig. 1C and Fig. 3A. Analysis of data in panels E-I was limited to the field of view on the axon side near microchannels, denoted with the cyan rectangle. Scale bar is 50 μm (left) or 5 μm for enlarged areas. The enlarged area bound by the cyan square shows representative images of microfluidic devices from CTRL KD and MFN2 KD 14-16 DIV, post MitoTracker labeling, displaying somato-dendritic mitochondrial traffic into axons (MTG) and resident axonal mitochondria (MTR). **E**) Quantitation of the average size of resident axonal mitochondria (MTR positive) between the CTRL KD and MFN2 KD microfluidic devices. CTRL KD resident axonal mitochondria were larger (0.39 μm^2^ [0.37 – 0.42]) than axonal mitochondria from MFN2 KD microfluidic devices (0.33 μm^2^ [0.32 – 0.36]). Values are medians with 95% CI representing error, Mann-Whitney test U = 95.5, n_1_ = 23, n_2_ = 23, p < 0.0001. Charts in panels E and F are Tukey box and whisker plots from 3 independent experiments, each n represents a field of view from the axon chamber. **F**) Quantitation of the average aspect ratio (major axis/minor axis) of resident axonal mitochondria (MTR positive) between the CTRL KD and MFN2 KD microfluidic devices. CTRL KD resident axonal mitochondria were longer (2.42 a.u. [2.37 – 2.54]) than axonal mitochondria from MFN2 KD microfluidic devices (2.30 a.u. [2.20 – 2.40]). Values are medians with 95% CI representing error, Mann-Whitney test U = 163.5, n_1_ = 23, n_2_ = 23, p = 0.0258. **G**) As in Fig. 3B, a measure of newly trafficked axonal mitochondria at 4 hours post MitoTracker labeling, comparing CTRL KD and MFN2 KD. At 4 hours, newly trafficked mitochondria account for 17.2% [14.60% - 22.80%] of total mitochondria in CTRL KD microfluidic devices, and newly trafficked mitochondria account for 9.6% [7.70% - 11.00%] of total mitochondria in MFN2 KD microfluidic devices. Values are medians with 95% CI representing error, Mann-Whitney test U = 43.5, n_1_ = 23, n_2_ = 20, p < 0.0001. Charts in panels G-I are Tukey box and whisker plots, with each n representing a field of view from the axon chamber. **H**) As in Fig. 3C, a measure of the fraction of newly trafficked mitochondria that fuse with resident axonal mitochondria. In CTRL KD microfluidic devices, 54.1% [40.30% - 61.70%] of newly trafficked mitochondria fuse with resident axonal mitochondria at 4 hours; in MFN2 KD microfluidic devices, 63.7% [55.10% - 66.30%] do,. Values are medians with 95% CI representing error, Mann-Whitney test U = 131.5, n_1_ = 23, n_2_ = 20, p = 0.0156. **I**) As in Fig. 3D, this is a measure of resident axonal mitochondria complementation. In CTRL KD microfluidic devices, 11.2% [7.50% - 17.20%] of resident axonal mitochondria fuse with newly trafficked axonal mitochondria at 4 hours; in MFN2 KD microfluidic devices, 8.0% [3.03% - 9.40%] do. Values are medians with 95% CI representing error, Mann-Whitney test U = 132.5, n_1_ = 23, n_2_ = 20, p = 0.0167. **J**) The redox ratio was calculated for CTRL KD (black) or MFN2 KD (red) conditions as described in Materials and Methods, then normalized to the average redox ratio of the CTRL KD population of neurons. The normalized redox ratio values were 1.00 [0.95 – 1.05] (oxidized to reduced) for CTRL KD and 1.05 [1.03 – 1.11] (oxidized to reduced) for MFN2 KD conditions. Values are medians with 95% CI representing error, Mann-Whitney test U = 303.5, n_1_ = 31, n_2_ = 29, p = 0.0302.

**Figure 5:**
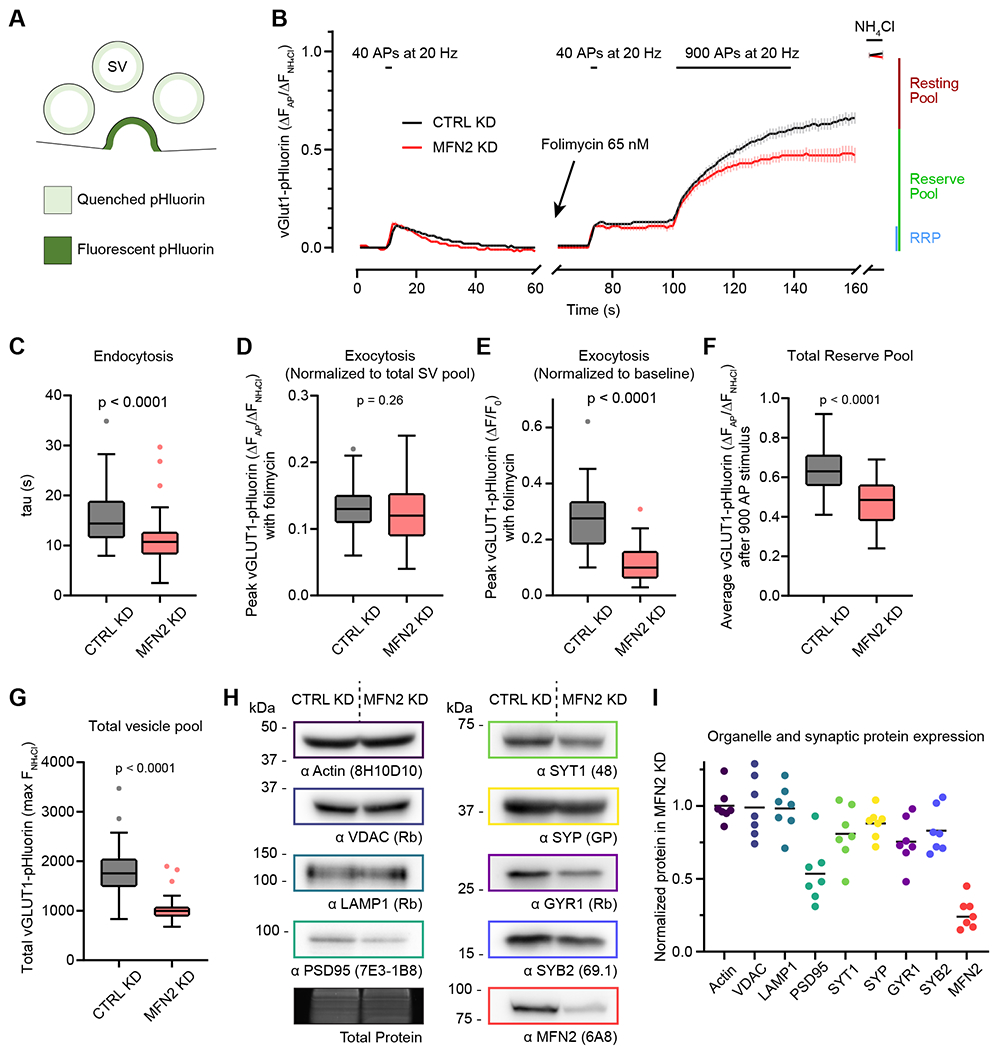
Mitofusin 2 supports the synaptic vesicle cycle **A**) Schematic of SV-targeted pHluorin during exocytosis. **B**) Average (± 95% CI) traces of vGlut1-pHluorin fluorescence intensity during the synaptic vesicle cycle from CTRL KD (black) and MFN2 KD (red) neurons. Neurons were stimulated with 40 action potentials (40 AP) at 20 Hz to drive the release of the readily releasable pool (RRP) and then allowed to recover. During recovery, the fluorescence decay represents endocytosis and vesicle reacidification. After recovery, folimycin was added to prevent vesicle reacidification (pHluorin quenching), enabling the measurement of pure exocytosis. Forty AP were used again to drive the release of the RRP; the neurons were then maximally stimulated with 900 AP at 20 Hz to trigger exocytosis of the entire RP of vesicles. After stimulation, NH_4_Cl was perfused onto the sample to unquench all synaptic pHluorin signal and measure the total vGlut1-pHluorin signal. Traces normalized to maximum NH_4_Cl signal ((F – F_0_)/(F_max_ – F_0_)), n > 45 separate boutons from 3 independent experiments. **C**) Rate of endocytosis (τ) determined from the vGlut1-pHluorin fluorescence decay after 40 AP stimulation. The median value with 95% CI intervals representing error is 14.39 s [13.17 – 17.85] for CTRL KD (black) boutons and 10.73 s [9.58 – 11.94] for MFN2 KD (red) boutons, Mann-Whitney test U = 474.5, n_1_ = 47, n_2_ = 45, p < 0.0001. Charts in panels C-G are Tukey box and whisker plots quantified from acquired traces in 5B, with n from 3 independent experiments. Each n represents a single bouton that was selected randomly after verification that the bouton stayed in focus and did not drift during acquisition. **D**) The RRP fraction as determined from the vGlut1-pHluorin fluorescence peak after a 40 AP stimulation, incubation with 65 nM folimycin, and normalization to total vesicle pool (NH_4_Cl bath). The median value with 95% CI intervals representing error is 13% [11% – 14%] for CTRL KD (black) boutons and 12% [10% – 13%] for MFN2 KD (red) boutons, Mann-Whitney test U = 913, n_1_ = 47, n_2_ = 45, p = 0.259. **E**) The RRP as assayed by vGlut1-pHluorin fluorescence peak after a 40 AP stimulation with folimycin but without NH_4_Cl normalization. The median value with 95% CI intervals representing error is 0.28 ∆F/F_0_ [0.22 – 0.31] for CTRL KD boutons and 0.10 ∆F/F_0_ [0.07 – 0.14] for MFN2 KD boutons, Mann-Whitney test U = 182, n_1_ = 47, n_2_ = 45, p < 0.0001. **F**) Total RP fraction assayed measuring the vGlut1-pHluorin fluorescence peak after incubation with 65 nM folimycin and stimulation with 940 AP. The median value with 95% CI intervals representing error is 63% [59% – 67%] for CTRL KD (black) boutons and 49% [41% – 55%] for MFN2 KD (red) boutons, Mann-Whitney test U = 323.5, n_1_ = 47, n_2_ = 45, p < 0.0001. **G**) A measure of the total vesicle pool as assayed by vGlut1-pHluorin fluorescence during NH_4_Cl perfusion. The median value with 95% CI intervals representing error is 1755 arbitrary fluorescence units (AFU) [1642 – 1954] for CTRL KD (black) boutons and 1002 AFU [952 – 1018] for MFN2 KD (red) boutons, Mann-Whitney test U = 124.5, n_1_ = 47, n_2_ = 45, p < 0.0001. **H**) Representative immunoblots used to quantify the indicated synaptic and organelle protein levels in CTRL KD and MFN2 KD neurons. Protein detected and antibody used (clone if monoclonal, species if polyclonal) are listed below the blots, and total protein was assayed by using Lumitein (Biotium) for every condition, including repeated lanes. **I**) Quantitation of protein levels in MFN2 KD samples, normalized to CTRL KD and total protein. Median values with 95% CI intervals representing error are 97% [86 – 124] for actin, 93% [74 – 129] for VDAC, 101% [71 – 121] for LAMP1, 48% [31 – 93] for PSD95, 79% [48 – 104] for SYT1, 90% [72 – 104] for SYP, 73% [48 – 98] for GYR1, 81% [67 – 106] for SYB2, and 24% [15 – 45] for MFN2. The p-values were calculated using Kruskal–Wallis test H = 35.39, n_1_ = 7, n_2_ = 7, n_3_ = 7, n_4_ = 7, n_5_ = 7, n_6_ = 7, n_7_ = 7, p < 0.0001. Following-up multiple comparisons by applying Dunn’s correction resulted in the following p-values: actin vs. PSD95 p = 0.0064 and actin vs. MFN2 p < 0.0001. Sample n are from 7 independent trials.

**Figure 6:**
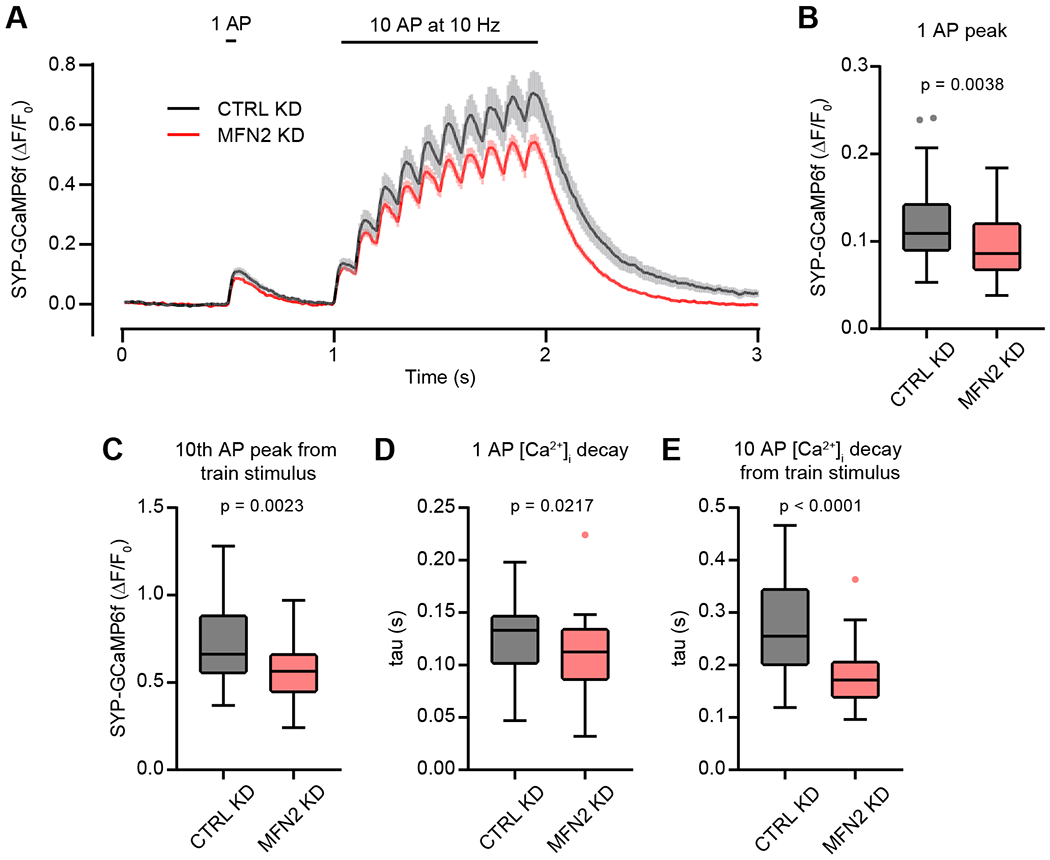
Mitofusin 2 supports presynaptic Ca^2+^ homeostasis **A**) Average (± 95% CI) traces of synaptophysin-GCaMP6f (SYP-GCaMP6f) fluorescence intensity after a single AP at 0.5 s and a 10 Hz, 10 AP train at 1 s from CTRL KD (black) and MFN2 KD (red) neurons. Traces are ∆F/F_0_ with n_1_ = n_2_ = 36, from 3 independent experiments, each n represents a single bouton after verification that the bouton stayed in focus and did not drift during the recording. **B**) Single AP SYP-GCaMP6f ∆F/F_0_ peak from CTRL KD (black) and MFN2 KD (red) boutons; median values with 95% CI intervals representing error were 0.11 ∆F/F_0_ [0.09 – 0.14] and 0.09 ∆F/F_0_ [0.07 – 0.09] respectively. Mann-Whitney test U = 393.5, n_1_ = n_2_ = 36, p < 0.0038. Charts in panels B-E are Tukey box and whisker plots quantified from acquired traces in 6a. **C**) Ten AP train SYP-GCaMP6f ∆F/F_0_ peak from CTRL KD (black) and MFN2 KD (red) boutons; median values with 95% CI intervals representing error were 0.66 ∆F/F_0_ [0.59 – 0.77] and 0.56 ∆F/F_0_ [0.47 – 0.63], respectively; Mann-Whitney test U = 381, n_1_ = n_2_ = 36, p = 0.0023. **D**) Rate of presynaptic Ca^2+^ clearance (τ) determined from SYP-GCaMP6f fluorescence decay after 1 AP from CTRL KD (black) and MFN2 KD (red) boutons; median values with 95% CI intervals representing error were 0.13 s [0.12 – 0.14] and 0.11 s [0.10 – 0.13], respectively; Mann-Whitney test U = 445, n_1_ = n_2_ = 36, p = 0.0217. **E**) Rate of presynaptic Ca^2+^ clearance (τ) determined from SYP-GCaMP6f fluorescence decay after a 10 AP train at 10 Hz from CTRL KD (black) and MFN2 KD (red) boutons; median values with 95% CI intervals representing error were 0.26 s [0.22 – 0.30] and 0.17 s [0.14 – 0.20], respectively; Mann-Whitney test U = 244, n_1_ = n_2_ = 36, p < 0.0001.

**Figure 7:**
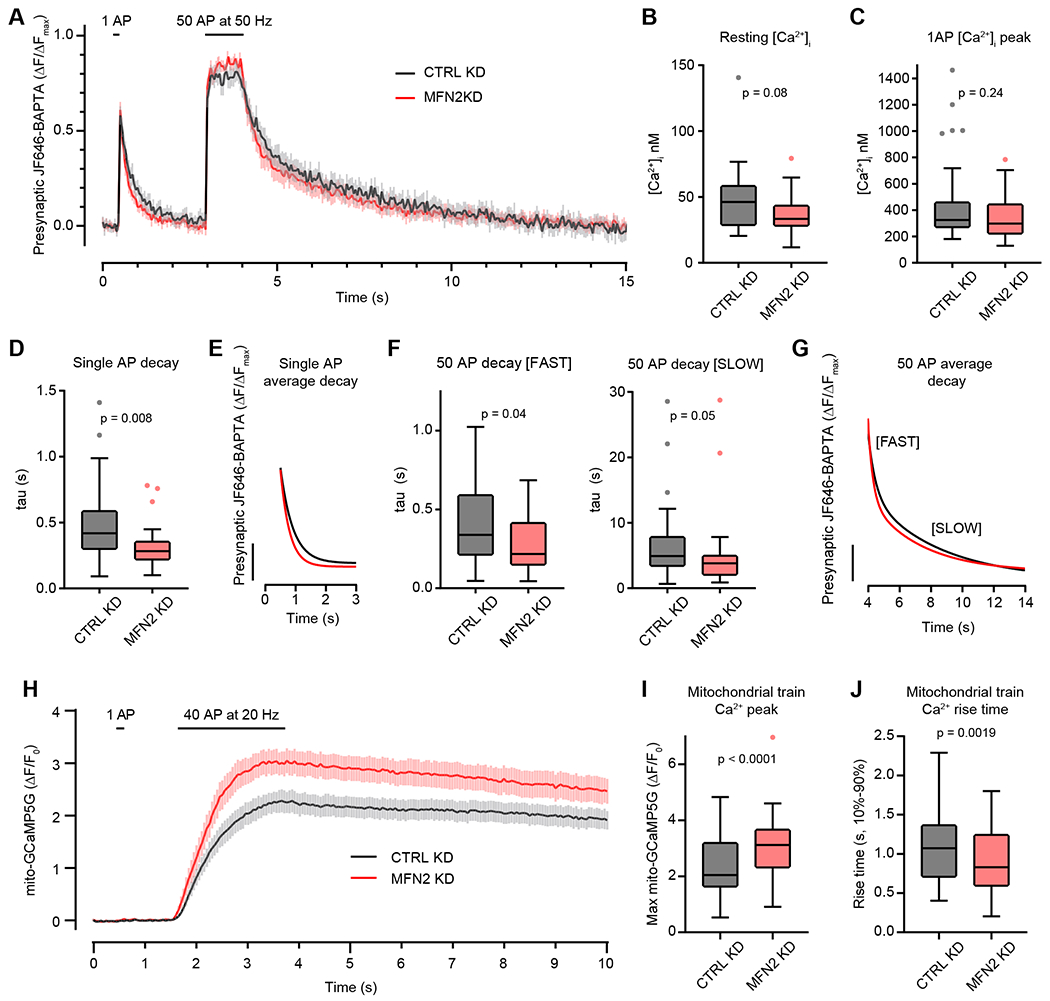
Mitofusin 2 disruption speeds presynaptic Ca^2+^ clearance by increasing mitochondrial Ca^2+^ uptake during neuronal activity **A**) Average ∆F/F_max_ (± 95% CI) traces of synaptophysin-HaloTag (SYP-HT)–bound HTL-JF646-BAPTA fluorescence intensity during electrical stimulation from CTRL KD (black) and MFN2 KD (red) neurons. Neurons were stimulated with 1 AP to quantitate calcium (Ca^2+^) influx. After 2.5 seconds, the neurons were maximally stimulated by 50 AP at 50 Hz to saturate the HTL-JF646-BAPTA and to calculate absolute Ca^2+^ concentration as described in Materials and methods. Traces are from n_1_ = n_2_ = 30 separate boutons, from 3 independent experiments. **B**) Resting presynaptic Ca^2+^ ([Ca^2+^]_i_) from CTRL KD (black) and MFN2 KD (red) boutons; median values were 46.2 nM [95% CI 32 – 54] and 33.4 nM [95% CI 29 – 37], respectively. Mann-Whitney test U = 330, n_1_ = n_2_ = 30, p = 0.0765. Charts in panels B-D and F are Tukey box and whisker plots quantified from the traces in panel A. **C**) Single AP peak [Ca^2+^]_i_ from CTRL KD (black) and MFN2 KD (red) boutons; median values were 325.5 nM [95% CI 282 – 380] and 298.1 nM [95% CI 235 – 381.4], respectively. Mann-Whitney test U = 370, n_1_ = n_2_ = 30, p = 0.2403. **D**) Single AP [Ca^2+^]_i_ τ from CTRL KD (black) and MFN2 KD (red) boutons. Averaged traces were well fitted to single-exponential functions, and median values were 419 ms [95% CI 372 – 540] and 283 ms [95% CI 242 – 312] for CTRL KD and MFN2 KD, respectively. Mann-Whitney test U = 272.5, n_1_ = n_2_ = 30, p = 0.0081. **E**) Single-exponential best fit curves to average CTRL KD (black) and MFN2 KD (red) [Ca^2+^]_i_ decay traces from 1 AP stimulation in panel a. **F**) A 50 AP–stimuli train (50 AP at 50 Hz) [Ca^2+^]_i_ τ from CTRL KD (black) and MFN2 KD (red) boutons. Averaged traces were well fitted with double-exponential functions, and median values for the [FAST] component were 339 ms [95% CI 250 – 513] and 218 ms [95% CI 165 – 361] for CTRL KD (black) and MFN2 KD (red) respectively. Mann-Whitney test U = 246.5, n_1_ = 26, n_2_ = 28, (note: some traces could not be fit by a double exponential), p = 0.0417. Median values for the [SLOW] component were 4.9 s [95% CI 3.5 – 7.1] and 3.8 s [95% CI 2.3 – 4.5] for CTRL KD (black) and MFN2 KD (red) respectively. Mann-Whitney test U = 250, n_1_ = 26, n_2_ = 28,p = 0.0489. **G**) Double-exponential best fit curves for the average CTRL KD (black) and MFN2 KD (red) [Ca^2+^]_i_ decay traces from 50 AP in 7A. **H**) Average ∆F/F_0_ (± 95% CI) fluorescence intensity traces from axonal mitochondria targeted with GCaMP5G to the mitochondrial matrix (mito-GCaMP5G) during electrical stimulation from CTRL KD (black) and MFN2 KD (red) neurons. Neurons were stimulated with 1 AP at 0.5 s followed by a train stimulus of 40 AP at 20 Hz at 1.5 s. Note: 1 AP did not cause robust mitochondrial Ca^2+^ uptake in presynaptic mitochondria. Traces are from n_1_ = 92, n_2_ = 77, from 3 independent experiments, with n representing individual axonal mitochondria. **I**) Peak mitochondrial Ca^2+^ (mito-GCaMP5g ∆F/F_0_) after train (40 AP at 20 Hz) stimulation from CTRL KD (black) and MFN2 KD (red) boutons, median values were 2.04 ∆F/F_0_ [95% CI 1.81 – 2.61] and 3.12 ∆F/F_0_ [95% CI 2.86 – 3.44], respectively. Mann-Whitney test U = 2147, n_1_ = 92, n_2_ = 77, p < 0.0001. Charts in panels I-J are Tukey box and whisker plots quantified from acquired traces in panel H. **J**) Mitochondrial Ca^2+^ rise time (10% to 90%, s) during train stimulation from CTRL KD (black) and MFN2 KD (red) boutons; median values were 1.07 s [95% CI 0.88 – 1.20] and 0.83 s [95% CI 0.67 – 0.94], respectively. Mann-Whitney test U = 2562, n_1_ = 92, n_2_ = 77, p = 0.0019.

Primary rat hippocampal cultures were transiently transfected with pEF cyto-cmCh and pEF mito-cGFP (matrix) (Fig. 1a), pEF mito-PAcGFP (Fig. 1b), pEGFP-N1-mt-ro1GFP (Fig. 2a–f), or pCAG mito-GCaMP5G (Fig. 7h–j) at 2-5 DIV using the Ca^2+^ Phosphate protocol (Jordan and Wurm, 2004). For microfluidic experiments, neurons were transfected or transduced in the soma chamber. Neurons in microfluidic devices were also labeled, with MitoTracker Green FM (Thermo Fisher Scientific) in the soma chamber and MitoTracker Red CMXRos (Thermo Fisher Scientific) in the axon chamber. Microfluidic chambers (soma and axon) were sequentially labeled, being sure to keep unstained chamber pressure high during staining to maintain fluidic isolation. Mass-dissociated neuronal cultures were transduced with vGLUT1-pHluorin, mito-roGFP, SYP-GCaMP6f, mito-GCaMP5G, and knockdown (control and MFN2) lentiviruses at 2-5 DIV. Neuronal cultures were moved to standard imaging media (i.e., extracellular fluid[ECF]) consisting of 140 mM NaCl, 5 mM KCl, 2 mM CaCl2, 2 mM MgCl2, 5.5 mM glucose, 20 mM HEPES (pH 7.3), B27 (Gibco), GlutaMAX (Gibco); loaded into the microscope; and maintained in a humidity-controlled chamber at near-physiological temperature (35°C). Cultures were imaged by using an Olympus FV1000 confocal microscope with a 60× 1.40 NA oil objective using fixed laser intensity and gain settings (Fig. 1a). Images were also captured on an Olympus IX83 inverted microscope equipped with a cellTIRF 4Line excitation system using an Olympus 60x/1.49 NA Apo N objective and an Orca Flash4.0 CMOS camera (Hamamatsu Photonics) running Metamorph software that was modified to run concurrently with Olympus 7.8.6.0 acquisition software from Molecular Devices (Fig. 1b–f; Fig. 2 to 7). Photoactivation experiments were conducted using a single, spot-focused, 405-nm laser on the CellTIRF system. Mitochondrial trafficking experiments were also conducted on the CellTIRF system by using wide-field acquisition mode. Image sequences analyzing mitochondrial motility were acquired with five-second intervals for a total of five minutes. Field electrical stimulation was triggered by a Grass SD9 stimulator run by Clampex 10.7.0.3 software (Molecular Devices) through a Digidata 1440A digitizer (Molecular Devices) to platinum parallel wires attached to a field-stimulation chamber (Warner Instruments; RC-49MFSH). Stimulator voltage was set to 90v as this reliably elicited Ca^2+^ transients at every bouton. For pHluorin and Ca^2+^ imaging experiments, D-AP5 (50 μM) (Abcam; ab120003), CNQX (20 μM) (Abcam; ab120044), and Picrotoxin (100 μM) (Tocris Bioscience; 1128) were added to the imaging media to prevent recurrent activity. **pHluorin imaging:** Synaptic vesicle exocytosis was monitored via change in fluorescence of presynaptic punctae from neurons transduced with the vGLUT1-pHluorin lentivirus. Images were acquired at 2×2 binning at 1 Hz for up to two minutes. For pHluorin imaging, the focal plane and field of view were found by live focusing during a test stimulation of 40 stimuli at 20 Hz. Neurons were given at least a minute to recover, and the stimulus was repeated during an image acquisition time of one minute. Next, freshly made 65 nM folimycin (Tocris Bioscience) was added to the imaging well. The macrolide antibiotics bafilomycin and folimycin inhibit the function of vacuolar-type H+ -ATPase, preventing pHluorin quenching of recently endocytosed vesicles and allowing a measure of pure exocytosis (Sankaranarayanan and Ryan, 2001; Atasoy et al., 2008). Within a minute of folimycin addition, a two-minute image acquisition was initiated and contained another pulse of 40 stimuli at 20 Hz, with a 30-second break, then a 900-stimuli train at 20 Hz (Burrone et al., 2006). At the end of this imaging sequence, a solution containing 50 mM NH_4_Cl (replacing 50 mM NaCl in ECF) was perfused onto the sample, dequenching all pHluorin (Miesenbock et al., 1998). **Ca^2+^ imaging:** Synaptic Ca^2+^ transients were recorded as synaptic fluorescence change from neurons transduced with synaptophysin-GCaMP6f (SYP-GCaMP6f) (Bradberry and Chapman, 2022) or synaptophysin-HaloTag (SYP-HT) reacted with HTL-JF646-BAPTA-AM (a gift from Dr Luke Lavis, Janelia Research Campus) (Deo et al., 2019). Presynaptic mitochondrial Ca^2+^ uptake during electrical activity was monitored in axonal mitochondria (axons identified by axon initial segment (AIS) staining and morphology) targeted with GCaMP5G (Kwon et al., 2016). Images were acquired at 2×2 binning at 20 Hz (50-ms exposure) for mito-GCaMP5G and SYP-HT/JF646-BAPTA for up to 20 seconds. SYP-GCaMP6f was recorded at 100 Hz (10-ms exposure). For SYP-HT/JF646-BAPTA, neurons were field-stimulated with a single stimulus with a break for 2.5 s, then a 50-stimuli train at 50 Hz was given to saturate the indicator. For SYP-GCaMP6f, neurons were field-stimulated with a single stimulus with a break for 0.5 s followed by a 10-stimuli train at 10 Hz. For mito-GCaMP5G, neurons were field-stimulated with a single stimulus with a break for 1.0 s followed by a 40-stimuli train at 20 Hz.

### Image Quantification

Mitochondrial motility (Fig. 1 and Fig. 2) was analyzed by using Imaris 8.3 (Bitplane). Motile mitochondria were defined as having at least 3 consecutive 15-s displacements during an image series. Mitochondrial redox ratio was calculated by using Fiji (NIH) (Schindelin et al., 2012) as described previously (Vevea et al., 2013). The roGFP redox ratio was normalized to the average ratio from the population of mitochondria of each timeseries. For roGFP range experiments, roGFP was normalized to initial values (paired recordings H_2_O_2_/DTT) or to CTRL (rotenone/antimycin A). Mitochondrial trafficking and fusion in axons was quantified by using the Just Another Colocalization Plugin (JACoP) (Bolte and Cordelieres, 2006) for Fiji (Fig. 3b–d and Fig. 4e–i). The pHluorin analysis was done by selecting responding ROIs in FIJI and measuring the fluorescence intensity change over time. These data were copied and imported into AxoGraph X 1.7.2 (AxoGraph Scientific), where traces were baseline-subtracted and normalized to pHluorin’s peak fluorescence intensity during NH_4_Cl perfusion via the formula (F – F_0_)/(F_max_ – F_0_). These traces were then analyzed in AxoGraph for endocytic τ (pHluorin signal decay after stimulus without folimycin), peak changes in fluorescence (exocytosis, with and without folimycin), and average fluorescence after 900 action potential (AP)–stimuli train but before NH_4_Cl application, which represents the total synaptic vesicle reserve pool (with folimycin). Ca^2+^ image quantitation was handled similarly to pHluorin analysis. Responding ROIs were identified, and fluorescence intensity was measured over time by using Fiji.

For SYP-GCaMP6f and mito-GCaMP5G imaging, the change in fluorescence over the initial fluorescence was calculated in AxoGraph, using the 0.5-s baseline as the initial fluorescence. This ∆F/F_0_ is a relative readout of compartment Ca^2+^ changes. For the HTL-JF646-BAPTA indicator, traces were baseline-subtracted and normalized to signal during the 50-AP train by calculating (F – F_0_)/(F_max_ – F_0_) in AxoGraph. The decays of [Ca^2+^]_i_ following the indicated stimulus are reported as τ values. The fluorescence decays after 1 AP were well fitted to a single-component exponential, and the decays after the train were well fitted to a two-component exponential. Absolute [Ca^2+^]_i_ was quantitated as described previously (Bradberry and Chapman, 2022). Briefly, the sensor was saturated (F_max_) with intense stimuli (Maravall et al., 2000), and published reports of K_d_ (140 nM), R_f_ (5.5), and Hill coefficient (n) (1) (Deo et al., 2019) were used to approximate [Ca^2+^]_i_ according to equations derived in (Maravall et al., 2000; de Juan-Sanz et al., 2017). The following equation was used to calculate [Ca^2+^]_i_ in the presynapse:

$${\left[{\text{Ca}}^{\text{2+}}\right]}_{\text{i}}=K_{d}{\left(\frac{\frac{F}{F_{max}}-\frac{1}{R_{f}}}{1-\frac{F}{F_{max}}}\right)}^{1/n}$$

where [Ca^2+^]_i_ represents internal calcium concentration, F is resting fluorescence, F_max_ is maximal fluorescence during indicator saturation, R_f_ is dynamic range, K_d_ is the dissociation constant, and n is the Hill coefficient. All measurements were summarized in Excel (Microsoft) and imported into GraphPad Prism 9.3.1 (GraphPad Software Inc.) for statistical analysis and graph production.

### Immunoblot protocol

Immunoblots were performed as described previously (Vevea and Chapman, 2020) but were imaged on a ChemiDoc MP Imaging System (Biorad, 17001402). Primary and secondary antibodies are listed in the key resource table along with identifiers and dilutions used.

### Chemicals

Chemicals are listed in the key resource table. All chemicals were resuspended and stored per manufacturer guidelines. Folimycin was ordered prior to use, stored dry at −20°C in original packaging, and resuspended the week of the experiments. Aliquots were frozen at −20°C and used within a week after resuspension.

### Statistics

Exact values from experiments and analysis, including the number of data points (n) and trials (i.e., biological replicates) for each experiment are listed in the figure legends. GraphPad Prism 9.3.1 (GraphPad Software Inc.) was used for statistical analysis. When appropriate, data are displayed as Tukey box and whisker plots. Most data were not normally distributed, so nonparametric tests (i.e., Mann-Whitney U tests) were used throughout. No a priori power analysis was completed before experimentation; however, to estimate sample size, a nomogram for sample size, effect size, and power was consulted (Serdar et al., 2021). For our experimental planning, we generally prefer power = 0.8 and confidence = 0.05. Then, we estimate the standardized difference to arrive at a rough sample size result. For initial mitochondrial size and trafficking parameters (Fig. 1), we wanted to ensure we uncovered a small to medium effect size, so we chose a standardized difference of approximately 0.4. This results in a required sample size of ~150-200. Finding a medium to large difference in these experiments, we adjusted our calculation and lowered our subsequent sample size for the experiments shown in Fig. 2. For sample size calculation in microfluidic experiments summarized in Fig. 4, we relied on the results from Fig. 3. For biosensor experiments (Fig. 5–7), we chose a standardized difference = 1, representing a large difference expectation, and resulting in an estimated sample size of ~30. For presynaptic biosensor image analysis, many boutons were imaged. The n that was quantified was 1) randomly selected for analysis and was checked only for a lack of x, y, and z drift during imaging; and 2) evenly dispersed between different image sets and biological replicates.

## Results

### Asymmetric trafficking of mitochondria in axons

The existence of axonal QC mechanisms regulating the asymmetric traffic of mitochondria remains the subject of debate. In unstressed axons, there is evidence both for (Miller and Sheetz, 2004; Mandal et al., 2021) and against (Verburg and Hollenbeck, 2008; Suzuki et al., 2018) mitochondrial trafficking asymmetry. There may not be a need for a trafficking QC mechanism in axons if mitochondrial biogenesis (Amiri and Hollenbeck, 2008; Kuzniewska et al., 2020) and degradation (mitophagy) (Ashrafi et al., 2014) occur locally. However, local biogenesis appears limited, and axonal mitophagy seems to occur only during extreme (likely non-physiological) stress. Importantly, increased mitochondrial retrograde traffic has been reported with application of low (nM) concentrations of mitochondrial toxins (Cai et al., 2012; Lin et al., 2017) or, in specific disease models (Ebrahimi-Fakhari et al., 2016; Zheng et al., 2019). We also note that several mitochondrial proteins are exceptionally long-lived (Bomba-Warczak et al., 2021), arguing against whole organelle degradation, and mitophagy might be completely dispensable for mitochondrial homeostasis in axons (Lin et al., 2021). The phenomenon of mitochondrial trafficking asymmetry appears to be evolutionarily conserved, as yeast (McFaline-Figueroa et al., 2011; Higuchi et al., 2013) and stem cells (Katajisto et al., 2015) can segregate low- from high-functioning mitochondria via a QC mechanism that is critical for replicative lifespan or maintaining stemness. Trafficking mitochondria in axons is expected to be important for axonal function and neuronal health because altered mitodynamics are found in many, if not all, neurodegenerative diseases and disease models examined, as well as for sporadic cases (Chen and Chan, 2009; Schon and Przedborski, 2011), but see also (Area-Gomez et al., 2019).

Here, we used dissociated rat hippocampal neurons to study axonal mitochondrial morphology and trafficking parameters. Before focusing on axonal mitochondria, we first corroborated morphological differences among mitochondria in the soma, dendrite (post-synaptic), and axonal (pre-synaptic) compartments (Figure 1A) (Lewis et al., 2018; Faitg et al., 2021). Dendrites have spines and a large tapering shaft diameter; axons are smooth, do not taper, and have a smaller diameter (Craig and Banker, 1994). Clear dendritic (right neurite) and axonal (left neurite) morphological characteristics are revealed by the cytosolic mCherry signal (Figure 1A, magenta box). Using mitochondrial targeted green fluorescent protein (mito-cGFP) (Figure 1A, green box), we confirmed that mitochondria in dendrites are long and overlapping, but axonal mitochondria are shorter and evenly spaced apart (Lewis et al., 2018; Faitg et al., 2021). We then examined trafficking using mitochondrial-targeted photo-activatable GFP (PA-GFP) (Patterson and Lippincott-Schwartz, 2002). Photo-activation of the soma allowed us to monitor new mitochondrial trafficking events to neurites (Figure 1B). Mitochondria that entered the axon were small and unitary and showed processive movement throughout the image series (30 min). In contrast, mitochondria entered dendrites slowly and did not traffic far, appearing to immediately fuse with the dendritic mitochondrial network, similar to previous observations (Overly et al., 1996). To further examine mitochondrial trafficking in the axon, we cultured primary neurons in microfluidic devices that separate axons from dendrites and cell bodies (Figure 1C; Movie 1). Microfluidic isolation enabled axon vs. somato-dendritic mitochondria counterstaining using different MitoTracker dyes. We then imaged axons in the middle of the 450-micron microfluidic channel to monitor retrograde (defined as MitoTracker Red +; MTR+) and anterograde (defined as MitoTracker Green +; MTG+) events. We found that anterograde-trafficked mitochondria were longer (Figure 1D) and more processive (Figure 1E) than retrograde-trafficked mitochondria; moreover, the average speed of anterograde mitochondria was greater (Figure 1F). Together, these data establish the existence of mitochondrial trafficking asymmetry in axons, which may be related to an organelle QC mechanism.

### Anterograde-trafficked mitochondria are more reduced and complement resident axonal mitochondria

We next examined the redox status of axonal mitochondria using microfluidic devices and mitochondrial-targeted redox-sensitive GFP (mito-roGFP). Mito-roGFP equilibrates with the mitochondrial matrix thiol/disulfide redox system and is an indirect reporter of any changes in reactive oxygen species (ROS) (Dooley et al., 2004; Hanson et al., 2004). The relative redox status of the mitochondrial matrix is used as a proxy for fitness of the organelle, with a more-reduced ratio indicating better fitness (McFaline-Figueroa et al., 2011). Relatively high mitochondrial ROS is a marker for organelle dysfunction, as high amounts of ROS are created as a byproduct of a disrupted electron transport chain and can further exacerbate mitochondrial damage and organelle dysfunction (Balaban et al., 2005). ROS is so well regarded as a marker for mitochondrial fitness that many studies are centered around observing the behavior of axonal mitochondria when exposed to mitochondrial toxins that increase these species. Specifically, these studies analyzed mitochondrial morphology, trafficking, and function in axons after application of high, moderate, or low doses of mitochondrial specific toxins that target either the mitochondrial membrane potential $\left(\Delta \Psi_{\text{m}}\right)$ or components of the electron transport chain.

High doses of antimycin A (AA, >40 μM), a complex III inhibitor, either arrested mitochondrial transport (Wang et al., 2011) and induced axonal mitophagy (Ashrafi et al., 2014), or increased axonal retrograde flux of mitochondria (Miller and Sheetz, 2004). A high dose of paraquat (20 mM) specifically inhibits mitochondrial traffic in the axon, dependent on ROS (Liao et al., 2017). A moderate dose of carbonyl cyanide m-chlorophenyl hydrazone (CCCP, 10 μM), a $\Delta \Psi_{\text{m}}$ dissipating reagent, increased axonal retrograde traffic to the soma (Cai et al., 2012); yet, a high dose (1 mM) did not affect the retrograde flux of mitochondria (Miller and Sheetz, 2004). Low doses of AA (<10 nM) increased retrograde mitochondrial transport (Lin et al., 2017) or had no effect on motility but induced mitophagy in the soma (Evans and Holzbaur, 2020). During basal or unstressed conditions, anterograde- and retrograde-trafficked mitochondria are reported to correlate strongly (Miller and Sheetz, 2004) or not at all (Verburg and Hollenbeck, 2008) with $\Delta \Psi_{\text{m}}$, or ATP levels (Suzuki et al., 2018); however, inhibition of retrograde axonal traffic increased the number of damaged mitochondria in axons (Mandal et al., 2021).

Here, we limited our study of axonal mitochondria to basal or unstressed conditions. We looked for evidence that axons can sort mitochondria based on their redox status. To do this, we transfected mito-roGFP into neurons growing in microfluidic devices and imaged the axon chamber just above (in the figure) the microchannel for 5-min periods (Figure 2A, magenta box). Antero- and retrograde-trafficking mitochondria were identified based on persistent motility in relation to the microfluidic microchannel, and their lengths and relative redox ratios were quantified. Because axon mitochondrial traffic asymmetry has been controversial, we again measured mitochondrial length, and again found anterograde mitochondria to be longer (Figure 2B). The difference here is significant but slightly less so than in Figure 1d, possibly because this experiment does not contain a counter stain to unambiguously distinguish somato-dendritic–derived anterograde mitochondria from retrograde mitochondria. Additionally, we found that anterograde-trafficking mitochondria had a more-reduced mitochondrial matrix than either stationary- (ratios are normalized to stationary mitochondria) or retrograde-trafficking mitochondria did (Figure 2C).

When using fluorescent biosensors, it is important to be sure the experimental recordings fall within the dynamic range of the chosen indicator. To address this, we demonstrate the range of mito-roGFP in our experiments with 30-minute applications of oxidant (5 mM H_2_O_2_) or reductant (5 mM Dithiothreitol) (Figure 2D). These experiments show that the mito-roGFP probe functions within its dynamic range during the experiments shown in Figure 2C. We also inhibited the electron transport chain with mitochondrial toxins, which increase mitochondrial ROS, using rotenone and antimycin A, which are complex I and III inhibitors, respectively. After 24 hours, we recorded an approximately 30% increase in the mito-roGFP ratio, representing a mitochondrial matrix that was apparently more oxidized than one receiving a 30-minute treatment with H_2_O_2_ (Figure 2E).

During these time-lapse experiments from panels A-C, we identified several mitochondrial fusion events. In Figure 2F and Movie 2, we show a representative example of an anterograde-trafficking mitochondria fusing with a stationary axonal mitochondrion with a more-oxidized status. The fused mitochondrion moved a small distance, the redox ratio equilibrated, and then the mitochondrion divided again, resulting in two mitochondria with relatively reduced mitochondrial matrix. Redox ratio values and mitochondrial area are included in the table to the right of the image frames. These fusion events prompted us to re-examine mitochondrial axonal trafficking using MitoTracker dyes and microfluidic devices in the following series of experiments.

### Robust anterograde axonal mitochondrial trafficking complements the resident axonal mitochondrial network

We monitored the rate of anterograde-trafficked mitochondria and the rate of axonal mitochondrial fusion by labeling neurons grown in a microfluidic device as in Figure 1 (Figure 3A; Movie 3). After labeling, we monitored the rate of newly trafficked mitochondria into the axon chamber every hour for four hours by imaging at the cyan box shown in Figure 3A. Somato-dendritic mitochondria (MTG+) trafficked into the axon chamber at a rate of ~4% of the total mitochondrial signal (MTR+ plus MTG+ mitochondria) per hour (Figure 3B). Approximately half (40-60%) of these newly trafficked mitochondria fused with resident axonal mitochondria (Figure 3C). We also addressed the rate at which resident axonal mitochondria are complemented and found that they fuse (colocalize) with newly trafficked mitochondria (MTG+) at a rate of ~2% per hour (Figure 3D). This value is the complementation rate. These results demonstrate vigorous anterograde axonal mitochondrial traffic (4% of total axonal mitochondrial mass per hour), which serves to complement the resident mitochondrial network in the form of fusion (~50% fusion rate). Next, we sought to disrupt this process and measure presynaptic function.

### Mitofusin 2 supports mitochondria morphology, anterograde trafficking, and mitochondrial complementation in axons

Mitochondrial fusion in vertebrates is mediated by the large outer membrane–bound GTPases mitofusin 1 (MFN1) and 2 (MFN2) (Chen et al., 2003). We disrupted mitochondrial fusion in neurons using a lentiviral-transduced shRNA to knockdown the main neuronal mitofusin isoform, MFN2 (Eura et al., 2003). We achieved stable and consistent MFN2 KD to ~20% that of control conditions (Figure 4A–B). In the soma of MFN2 KD neurons, mitochondria appeared swollen and less tubular than control knockdown cells (CTRL KD, scrambled shRNA) (Figure 4C). We then used microfluidic devices to label the soma side with MTG and the axon side with MTR as described in Figure 1, but this time we compared axonal mitochondrial traffic and fusion between CTRL KD and MFN2 KD neurons. As before, fields of view were collected on the axon side near the microchannel opening, indicated by the cyan box (Figure 4D); enlarged regions of interest showing representative CTRL KD and MFN2 KD axonal mitochondria, are shown to the right. We first compared shape descriptors, finding that axonal mitochondria from MFN2 KD microfluidic devices were smaller (Figure 4E) and shorter (Figure 4F) than CTRL KD cells, as measured by individual mitochondrial area (μm^2^) and aspect ratio (major axis/minor axis), respectively. Next, we examined mitochondrial trafficking and fusion in axons, as described in Figure 3B–D, at a 4-hour timepoint. For CTRL KD cells, the values of newly trafficked axonal mitochondria as a percentage of total mitochondria (Figure 4G), the fraction of newly trafficked mitochondria undergoing fusion (Figure 4H), and the fraction of resident mitochondria undergoing fusion in the axon at the 4-hour timepoint (Figure 4I) are comparable to values quantified for Figure 3, which depicts untransduced cells. As expected, untransduced control neurons and transduced CTRL KD neurons in microfluidic devices are comparable, further documenting that the methodology and measurements of mitochondria are robust and reproducible. Comparing the above parameters for CTRL KD and MFN2 KD axonal mitochondria, we observed decreased anterograde mitochondrial traffic (new axonal mitochondria identified by MTG) (Figure 4G), an increased rate of mitochondrial fusion of newly trafficked mitochondria (Figure 4H), but an overall decrease in total axonal mitochondrial fusion in MFN2 KD axons (Figure 4I). We also assayed neuronal mitochondrial redox ratios, comparing CTRL KD to MFN2 KD cells, and found that MFN2 KD neurons had a slightly more oxidized mitochondrial matrix (elevated ~5%) (Figure 4J).

MFN2 influences mitochondrial trafficking in zebrafish axons (Chapman et al., 2013), as well as in the axons of rodent dorsal root ganglion (DRG) sensory neurons (Misko et al., 2010) and human embryonic stem cell–derived spinal motor neurons (Mou et al., 2021). Here, in primary rodent hippocampal axons, we observed decreased anterograde mitochondrial traffic and an overall decreased amount of mitochondrial fusion to resident axonal mitochondria. Surprisingly, the limited number of mitochondria that successfully trafficked into the axon chamber appeared to exhibit no defects in fusion. In fact, we document a slight increase in fusion rate relative to CTRL KD conditions. This may be a sign of early QC during mitochondria soma egress; neurons may select for fusogenic mitochondria. In our MFN2 KD conditions, this would appear as an overall decrease in mitochondrial anterograde traffic followed by a normal fusion rate for newly trafficked axonal mitochondria, which is what we observed.

### Mitofusin 2 supports the synaptic vesicle cycle

We next determined whether loss of mitochondrial fusion influences the SV cycle using an SV-targeted super-ecliptic pHluorin (vGlut1-pHluorin) (Voglmaier et al., 2006) as a read-out for exocytosis and endocytosis. The phluorin tag is a pH-sensitive GFP mutant with a pKa of ~7.1 (Sankaranarayanan et al., 2000). Upon SV exocytosis, pHluorin dequenches (i.e., the fluorescence increases); then, upon endocytosis and reacidification, it requenches (i.e., the fluorescence decreases). We investigated the SV cycle by monitoring the fluorescence changes from vGlut1-pHluorin in CTRL and MFN KD neurons; an illustration of the vGlut1-pHluorin response upon SV exocytosis is shown in Figure 5A. We measured the rate of endocytosis and the size of the readily releasable pool (RRP), reserve pool (RP), and total SV pool using established protocols (Li et al., 2005). Briefly, 40 action potentials (40 AP) drive the release of the RRP, and 900 AP drive the release of the RP. Endocytosis occurs during exocytosis; therefore, to accurately measure exocytosis, a V-ATPase inhibitor (e.g., folimycin) is required to inhibit SV reacidification. Finally, bath application of NH_4_Cl was used to dequench all vGlut1-pHluorin, and this value was used to normalize the pHluorin signals at each synapse. The average traces for RRP and RP depleting stimuli are shown in Figure 5B. First, we found that the rate of endocytosis (tau; τ) was faster at MFN2 KD synapses than at CTRL KD synapses (Figure 5C). The NH_4_Cl normalized (total SV pool normalization) RRP with folimycin (Figure 5D) was unchanged between CTRL KD and MFN2 KD synapses; however, the non-normalized RRP from MFN2 KD neurons was significantly lower (Figure 5E). Indeed, the NH_4_Cl normalized reserve pool fraction was also smaller (Figure 5F), as was the total fluorescence during bath application of NH_4_Cl (Figure 5G), suggesting a major reduction in the size of the total SV pool in MFN2 KD neurons.

The vGlut1-pHluorin results led us to investigate protein levels of select organelle markers and key presynaptic protein levels via immunoblot analysis. We compared CTRL and MFN2 KD neuron protein levels by examining markers for the cytoskeleton (actin), mitochondria (voltage-dependent anion channel, VDAC), lysosomes (lysosome-associated membrane protein 1, LAMP1), as well as markers of synaptic vesicle proteins (synaptotagmin 1, SYT1; synaptophysin, SYP; synaptogyrin 1, GYR1; synaptobrevin 2, SYB2), and a postsynaptic marker (post-synaptic density 95, PSD95) (Figure 5H). Protein levels of actin, VDAC, and LAMP1 were unaltered in MFN2 KD neurons; however, presynaptic protein markers were decreased by ~25%, and PSD95 was decreased by ~50%. In these trials, MFN2 was decreased by ~75% (Figure 5I). These experiments demonstrate that mitochondrial fusion is necessary for normal rates of endocytosis, total amounts of exocytosis, mobilization of the RP during intense stimulation, and the total size of the SV pool.

### Mitofusin 2 supports presynaptic Ca^2+^ homeostasis

To gain insight into the pHluorin responses, we next investigated presynaptic Ca^2+^ following a single AP, as well as a train of APs, using presynaptically targeted GcaMP6f (SYP-GcaMP6f) (Chen et al., 2013). Neurons transduced with SYP-GcaMP6f showed dim fluorescent puncta corresponding to presynaptic clusters of SVs, which increased in fluorescence during electrical stimulation. For this analysis, we stimulated with one depolarization (1 AP) at 0.5 s before stimulating with 10 AP at 10 Hz at the 1 s time-mark; the image series ends at 3 s, after the indicator has returned to baseline. The average traces of ∆F/F_0_ SYP-GCaMP6f are shown in Figure 6A. First, we quantified the peak changes in ∆F/F_0_ SYP-GCaMP6f and found that the peak signal (peak presynaptic Ca^2+^) in MFN2 KD neurons was lower following a single AP (Figure 6B) or 10 APs at 10 Hz (Figure 6C), as compared to CTRL KD neurons, revealing lower presynaptic [Ca^2+^]_i_ during electrical activity in MFN2 KD conditions. Then we fitted the post-stimulus ∆F/F_0_ SYP-GCaMP6f fluorescence to single exponential decay functions to find the rate of presynaptic Ca^2+^ clearance (tau; τ). We detected a modest increase in decay rate following a single AP (Figure 6D) and a much-faster Ca^2+^ decay following a train stimulation (10 AP at 10 Hz) (Figure 6E) in MFN2 KD neurons compared to CTRL KD neurons. It is well accepted that presynaptic mitochondria influence exocytosis and Ca^2+^ (Tang and Zucker, 1997; Billups and Forsythe, 2002; Kwon et al., 2016; Vaccaro et al., 2017), so we examined our Ca^2+^ (GCaMP6f) and exocytosis (pHluorin) datasets for evidence of two different populations, representing boutons with and without mitochondria. We did not observe segregation, suggesting overlap between these two groups. Overall, the presynaptic GCaMP6f findings suggest an impairment of Ca^2+^ influx and/or increased Ca^2+^ efflux from, or sequestration in, the presynaptic compartment, potentially explaining our pHluorin measurements.

### Mitofusin 2 disruption speeds presynaptic Ca^2+^ clearance by increasing mitochondrial Ca^2+^ uptake during neuronal activity

To gain further insight into altered presynaptic Ca^2+^ homeostasis during activity, we next used HTL-JF646-BAPTA-AM targeted to the presynapse by synaptophysin-HaloTag (SYP-HT) (Deo et al., 2019; Bradberry and Chapman, 2022). This probe exhibits faster “on” kinetics, allowing us to better probe Ca^2+^ influx; it also allowed us to calculate the presynaptic Ca^2+^ concentration ([Ca^2+^]_i_). With a stimulation paradigm of a single AP, followed by a short recovery and a sensor-saturating stimulation of 50 AP at 50 Hz, followed finally by a longer recovery, we calculated (described in Materials and Methods) the resting [Ca^2+^]_i_, [Ca^2+^]_i_ entry following 1 AP, as well as the [Ca^2+^]_i_ decay after 1 AP or after a saturating train of APs. Average traces from CTRL and MFN2 KD presynapses are shown in Figure 7A. We found that there was a trend toward lower resting [Ca^2+^]_i_ in the MFN2 KD condition (Figure 7B) and no difference in Ca^2+^ entry in response to 1 AP (Figure 7C). However, the [Ca^2+^]_i_ decay (τ) in the MFN2 KD condition was altered. Presynaptic [Ca^2+^]_i_ decay following 1 AP was well fitted by a single-exponential function, but decay following a 50 AP train required a double-exponential function. These observations agree well with those from previous studies that longer bursts of activity elicit a Ca^2+^ decay that is well fitted to a double-exponential (Tank et al., 1995; Koester and Sakmann, 2000; Zhang and Linden, 2012), with the second component mediated by mitochondrial Ca^2+^ flux (Werth and Thayer, 1994). Because this is a high-affinity Ca^2+^ sensor, the rate of [Ca^2+^]_i_ decay measured here is not reflective of the absolute clearance rate (Regehr and Atluri, 1995), but relative differences between conditions may still be informative, so we also quantified these. Following a single AP, [Ca^2+^]_i_ decreases faster in the MFN2 KD than in the CTRL KD condition (Figure 7D), with the best fit exponentials compared in Figure 7E. Following a 50-AP train, both time constants (τ) representing [Ca^2+^]_i_ decay also trend faster in the MFN2 KD condition (Figure 7F), with the best fit exponentials compared in Figure 7G. Interestingly, the τ from 1 AP is the same as the [FAST] τ from the train, suggesting that the same [Ca^2+^]_i_ decay mechanism governs both processes (compare values from Figure 7D to those from Figure 7F [FAST]).

After extensive examination of presynaptic [Ca^2+^]_i_ transients during activity, we became confident that there is little to no change in basal [Ca^2+^]_i_ and likely no change in [Ca^2+^]_i_ influx, but an increase in the rate of presynaptic [Ca^2+^]_i_ clearance. Mitochondria store Ca^2+^ and because we are perturbing mitochondria, we monitored [Ca^2+^]_mito_ uptake during activity to potentially explain the changes in [Ca^2+^]_i_ observed in the presynapses of MFN2 KD neurons. We probed [Ca^2+^]_mito_ by transfecting mito-GCaMP5G (Kwon et al., 2016) into neurons, identifying axonal mitochondria, and stimulating neurons with a single AP (1 AP) as well as an RRP-depleting stimulus train (40 AP at 20 Hz). Average traces from CTRL and MFN2 KD presynaptic mitochondria are shown in Figure 7H. We found that mitochondria in CTRL KD conditions did not accumulate [Ca^2+^]_mito_ following a single AP (n = 0/92); however, we did observe three instances of [Ca^2+^]_mito_ accumulation following a single AP in mitochondria from MFN2 KD presynapses (n = 3/77). The majority of mitochondria likely do not accumulate [Ca^2+^]_mito_ following a single AP because, as seen in Figure 7C, [Ca^2+^]_i_ reaches only approximately 300 nM, and the mitochondrial Ca^2+^ uniporter is known to have a low affinity (Szabadkai and Duchen, 2008) such that it drives the sequestration of Ca^2+^ only when local concentrations rise above ~500 nM (Rizzuto et al., 1992). Because peak [Ca^2+^]_i_ from a single AP is no different in CTRL conditions than in MFN2 KD conditions (Figure 7C), the rare ability for MFN2 KD mitochondria to accumulate Ca^2+^ was unexpected. Upon an RRP-depleting stimulation (40 AP at 20 Hz), mitochondria in both KD conditions accumulated Ca^2+^. However, MFN2 KD presynaptic mitochondria accumulated approximately 50% more Ca^2+^ (Figure 7I) and did so at a faster rate (Figure 7J). Presynaptic mitochondria from MFN2 KD neurons are better than mitochondria from CTRL KD conditions at accumulating [Ca^2+^]_mito_ during neuronal activity. We expected MFN2 KD to somehow limit mitochondrial function; however, these mitochondria have a clearly enhanced ability to take up cytosolic Ca^2+^, which may be explained if mitochondria were physically closer to presynaptic Ca^2+^ channels. We expound on this possibility in the Discussion.

## Discussion

### Mitochondrial trafficking asymmetry in axons

In the current study, we document differences in mitochondrial trafficking between axons and dendrites, as well as trafficking asymmetry within axons, all under unstressed conditions. Anterograde axonal mitochondria are larger and relatively more reduced; retrograde axonal mitochondria are shorter and relatively more oxidized (Figure 1 and Figure 2). Importantly, these studies are done in mature, unchallenged, primary dissociated hippocampal cultures, and we use microfluidic devices and MitoTracker dyes to unambiguously define anterograde- and retrograde-moving axonal mitochondria. The mitochondria we observe in Figure 1, in the middle of the microchannel, have trafficked a minimum of 150 microns in a single direction. We believe this is the primary reason that we see such a clear distinction between anterograde and retrograde populations. Our data demonstrate a size (length) and biochemical (redox) trafficking asymmetry of mitochondria in the axon, which we interpret as evidence of an active QC mechanism. Indeed, during our axonal mito-roGFP trafficking experiments, we observed fusion events between relatively reduced anterograde trafficking mitochondria, and stationary, more-oxidized mitochondria. After fusion, their relative redox state appeared to rebalance, providing clear evidence of biochemical complementation.

### Role of mitochondrial fusion in maintaining the axonal mitochondrial network

Anterograde mitochondria that are larger and more reduced could either establish new mitochondrial outposts or fuse with resident, stationary axonal mitochondria. We again used microfluidic devices, but this time we examined mitochondrial content mixing in the axon chamber after two-color MitoTracker labeling. In these experiments, we found that resident axonal mitochondria fused with newly trafficked mitochondria at a rate of ~2% per hour (Figure 3). Importantly, ~50% of the newly trafficked axonal mitochondria (MTG+) fuse with the resident pool of axonal mitochondria. This means that half of the newly trafficked mitochondria serve to fuse with, and potentially complement, resident axonal mitochondria; the fate of the other half of the newly trafficked mitochondria is unclear. These experiments demonstrate the dynamic fusion properties of mitochondria within axons and suggest that continual mitochondrial fusion may be important to support the mitochondrial network and axon function (i.e., the SV cycle). Indeed, mitochondrial complementation (via fusion) suppresses mtDNA disease–associated alleles in various mouse tissues (Nakada et al., 2001), and loss of mitochondrial fusion increases mitochondrial heterogeneity and dysfunctional mitochondrial units in mouse embryonic fibroblasts (Chen et al., 2005). In INS1 and COS7 cells, fusion is selective and precedes fission events (Twig et al., 2008); in rat dorsal root ganglion neurons, fusion hyperpolarizes mitochondria and promotes ATP synthesis (Suzuki et al., 2018).

### Mitofusin 2 supports mitochondrial trafficking and fusion in axons to influence the SV cycle.

We again used neurons grown in microfluidic devices and labeled with MitoTracker dyes to examine the role of Mitofusin 2 (MFN2) in maintaining the axonal mitochondrial network. Altering the expression of the mitochondrial fusion or fission machinery will alter the morphology of mitochondria in axons (Amiri and Hollenbeck, 2008). Disrupting mitochondrial fission leads to loss of mitochondria in dopaminergic axons (Berthet et al., 2014), decreased neurite and synapse formation in primary neuronal cultures (Ishihara et al., 2009), and enhanced depression in acute hippocampal slices (Oettinghaus et al., 2016). Disrupting mutations in MFN2 infamously drive a progressive form of Charcot-Marie-Tooth syndrome (type 2A); these mutations decrease anterograde axonal mitochondrial traffic, thus altering distribution of these organelles (Baloh et al., 2007; Misko et al., 2010; Misko et al., 2012). Finally, suppressing MFN2 expression in human-induced pluripotent stem cell–derived neurons inhibits differentiation and synaptogenesis (Fang et al., 2016). Here, we found that KD of MFN2 reduces the size and total anterograde trafficking of axonal mitochondria, leading to decreases in the total number of mitochondria, and the total fusion of new mitochondria in axons. Interestingly, the few mitochondria in MFN2 KD neurons that successfully trafficked into the axon had no apparent defects in fusing with resident axonal mitochondria (Figure 4).

We then investigated the role of mitochondrial trafficking and fusion in supporting a key function of the axon, the SV cycle, by knocking down MFN2 and expressing SV-targeted pHluorin. We found that decreased mitochondrial trafficking and fusion through MFN2 KD leads to faster endocytosis, a reduction in the RRP (but no change in the normalized RRP due to smaller total vesicle pool size), difficulty in mobilizing the RP, and decreased expression of SV-associated proteins (Figure 5). Here we focused on disrupting fusion through MFN2 KD, but disrupting fission also inhibits the mobilization of the RP at *Drosophila* neuromuscular junctions (NMJ) (Verstreken et al., 2005), strongly suggesting that mitochondrial localization or morphology influence the SV cycle. However, additional studies in *Drosophila* suggest that although mitochondrial morphology is critical for localization and trafficking in the axon, it is dispensable for normal function of the neuron (Trevisan et al., 2018).

### The axonal mitochondrial network maintains presynaptic Ca^2+^

Mitochondria that lack a membrane potential fail to buffer presynaptic Ca^2+^, resulting in increased synaptic depression rates; this depression is completely rescued by supplementing the buffering of cytosolic Ca^2+^ (Billups and Forsythe, 2002). Similarly, impairing the ability of presynaptic mitochondria to sequester Ca^2+^, by decreasing the expression of the mitochondrial Ca^2+^ uniporter, enhanced synaptic release (Devine et al., 2022). Altering the size of mitochondria also changes their Ca^2+^ buffering capacity. Smaller mitochondria in MFN2 KD C2C12 myotubes buffer less Ca^2+^ and have lower resting [Ca^2+^]_i_ (Kowaltowski et al., 2019) and (Naon et al., 2016). In addition, disrupting mitochondrial fission in C2C12 myotubes (Kowaltowski et al., 2019) or in mouse pyramidal neurons (Lewis et al., 2018) enhances mitochondrial Ca^2+^ buffering capacity. The merging view is that mitochondria influence presynaptic Ca^2+^ transients to, in turn, modulate presynaptic properties [recently reviewed in (Datta and Jaiswal, 2021)]. Our pHluorin results prompted us to investigate presynaptic Ca^2+^, comparing CTRL KD to MFN2 KD conditions.

We first used a presynaptic targeted GCaMP6f (SYP-GCaMP6f) to monitor [Ca^2+^]_i_ during AP. GCaMP6f is widely utilized to monitor neuronal Ca^2+^ transients at the cell body scale and is reasonably well suited to also monitor presynaptic Ca^2+^ transients, with its high signal-to-noise ratio, large dynamic range, fast off-rate, and moderately fast on-rate (Chen et al., 2013). Using this sensor, we found evidence for decreased peak [Ca^2+^]_i_ in MFN2 KD presynapses following a single AP or using a 10-AP, 10-Hz train stimulation. The [Ca^2+^]_i_ decays were also faster in MFN2 KD conditions (Figure 6). To further study presynaptic [Ca^2+^]_i_, we utilized the recently developed far-red BAPTA–based indicator JF646-BAPTA-AM (Deo et al., 2019), which allowed us to calculate resting and peak presynaptic [Ca^2+^]_i_ following a single AP. This sensor has faster “on” kinetics than GCaMPs, yielding a more-accurate measure of Ca^2+^ influx kinetics. We found a trend toward decreased resting [Ca^2+^]_i_ and no difference between CTRL and MFN2 KD neuron Ca^2+^ influx from a single AP. We also found that Ca^2+^ transients in MFN2 KD neurons decayed faster than CTRL neurons after a single AP following a saturating train of stimuli (50 AP at 50 Hz) (Figure 7).

Mitochondria are a key Ca^2+^ buffer at the presynapse, and loss of this mitochondrial function shortens presynaptic Ca^2+^ transients in DRG neurons (Werth and Thayer, 1994). Indeed, loss (Marland et al., 2016) or decreased expression (Devine et al., 2022) of the mitochondrial Ca^2+^ uniporter, increases the rate of SV endocytosis in mouse hippocampal neurons; however, the role of Ca^2+^ in endocytosis is complex. Increased [Ca^2+^]_i_ either promotes (Neher and Zucker, 1993; Neves et al., 2001; Sankaranarayanan and Ryan, 2001; Wu et al., 2005; Balaji et al., 2008) or inhibits endocytosis (von Gersdorff and Matthews, 1994; Sankaranarayanan and Ryan, 2000; Leitz and Kavalali, 2011), see also a thorough review (Leitz and Kavalali, 2016). Therefore, we followed our Ca^2+^ journey into the mitochondrial matrix. We monitored matrix Ca^2+^ accumulation during a single AP and RRP-depleting trains of AP (40 AP at 20 Hz) using mito-GCaMP5G. We expected that mitochondria in MFN2 KD axons would be slightly dysfunctional and, therefore, have a decreased ability to import Ca^2+^, resulting in faster [Ca^2+^]_i_ clearance from the presynapse as subsequent delayed Ca^2+^ release from mitochondria would be reduced. However, we observed the opposite: axonal mitochondria from MFN2 KD neurons import ~50% more Ca^2+^ than their CTRL KD counterparts do. Although initially confounding, there is precedent in the literature for this, as ablation of MFN2 in cardiomyocytes results in mislocalization of mitochondria to the periphery (Kasahara et al., 2013) and loss of MFN2 in mouse embryonic fibroblasts results in enhanced mitochondrial Ca^2+^ sequestration during Ca^2+^ entry from the plasmalemma (Munoz et al., 2013; Naon et al., 2016). So, we interpret our results by concluding that MFN2 KD not only reduces mitochondrial anterograde axonal traffic and axonal mitochondrial fusion but also results in mitochondrial mislocalization, closer to the plasmalemma. There, they are exposed to higher Ca^2+^ concentrations allowing them to take up larger amounts of Ca^2+^. This presents an exciting hypothesis: mitochondrial Ca^2+^ overload may be the genesis of mitochondrial dysfunction in diseases such as Charcot-Marie-Tooth disease type 2A and subsequently allow a bolus of Ca^2+^ release in the axon, which may trigger axonal degeneration (Misko et al., 2012).

### Final thoughts

MFN2 is a large membrane protein with numerous cellular functions. Its original and most widely recognized function is to catalyze the homotypic fusion of outer mitochondrial membranes (Chen et al., 2003). It also either positively (de Brito and Scorrano, 2008; Naon et al., 2016) or negatively (Cosson et al., 2012; Filadi et al., 2015) regulates mitochondria-ER contact sites and may interact with motor adaptors in vertebrates (Misko et al., 2010; Misko et al., 2012). Therefore, the interpretation of phenotypes resulting from constitutive knockouts or even acute KD experiments, as done here, should be made with caution. Regardless, these experiments are essential to begin to understand the role of axonal mitochondrial QC and the physiology of the axon. Exciting questions about the fundamental signals that govern the maintenance and QC of the axonal mitochondrial network remain. A large body of research revealed that loss of Δψ_m_ marks a mitochondrion for degradation, making it unable to rejoin the network (Pickles et al., 2018), but what is the signal that identifies a mitochondrion for a complementation event? How are mitochondrial fusion and fission events linked? We note that MFN2 itself is sensitive to ROS, and this sensitivity may provide an elegant mechanism to activate the fusion machinery (Shutt et al., 2012; Mattie et al., 2018). Other important questions include: how are mitochondria selected for axonal versus dendritic egress from the soma? Is there a QC step at this early stage in trafficking? What are some of the damage markers that build up over time, and can they be influenced? These issues are important for understanding mitochondrial quality control mechanisms and how they support the SV cycle and neuronal health.

## Supplementary Material

Video 1 Movie 1: Asymmetric trafficking of mitochondria in axons
Example image sequence of background-subtracted MTG+ and MTR+ axonal mitochondria trafficking through the microchannel of a 450 μm–long microfluidic. The image sequence is an example from data collected for Figure 1C. Intervals between frames are 5 s each. Scale bar: 5 μm

Video 3 Movie 3: Robust anterograde axonal mitochondrial trafficking complements the resident axonal mitochondrial network.
Example image sequence of background-subtracted MTG+ and MTR+ axonal mitochondria trafficking in the axonal chamber 2 hours post-MitoTracker labeling. The image sequence is an example from data collected for Figure 3A. Intervals between frames are 5 s each. Scale bar: 5 μm

Video 2 Movie 2: Anterograde mitochondria are more reduced and complement resident axonal mitochondria.
Example timeseries from the axon chamber of a microfluidic device showing mitochondria expressing roGFP. The image sequence is the background-subtracted ratio of both emissions from roGFP. This is the full movie from Figure 2F, which shows select images. Fire LUT represents reduced (purple) or oxidized (white) signal. Intervals between frames are 5 s each. Scale bar: 5 μm

## Figures and Tables

**Table:** Key Resource Table

Reagent type (species) or resource	Designation	Source or reference	Identifiers	Additional information
biological sample (*Rattus norvegicus*)	Primary rat hippocampal neurons	Envigo	Sprague Dawley	
cell line (*Homo-sapiens*)	HEK293T, Kidney epithelial	ATCC	CRL-11268	
recombinant DNA reagent	FUGW (plasmid)	Addgene	Addgene plasmid # 14883; http://n2t.net/addgene:14883 ; RRID:Addgene_14883	Lentivirus backbone
recombinant DNA reagent	pEF-GFP (plasmid)	Addgene	Addgene plasmid # 11154; http://n2t.net/addgene:11154 ; RRID:Addgene_11154	pEF backbone
recombinant DNA reagent	pEF cyto-cmCh (plasmid)	This study	Addgene	Materials and methods
recombinant DNA reagent	pEF mito-cGFP (matrix) (plasmid)	This study	Addgene	Materials and methods
recombinant DNA reagent	pEF mito-PAcGFP (plasmid)	This study	Addgene	Materials and methods
recombinant DNA reagent	pF(UG) hSyn vGlut1 pHluorin (plasmid)	This study	Addgene	Materials and methods
recombinant DNA reagent	pEGFP-N1-mt-ro1GFP (plasmid)	Addgene	Addgene plasmid # 82407 ; http://n2t.net/addgene:82407 ; RRID:Addgene_82407	
recombinant DNA reagent	TRC2 pLKO.5-puro non-mammalian shRNA Control (plasmid)	Sigma	SHC202	CTRL KD
recombinant DNA reagent	pLKO.1 MFN2KD (plasmid)	Sigma	TRCN0000080612	MFN2 KD
recombinant DNA reagent	pF(UG) hSyn SYP-HaloTag (plasmid)	This study	Addgene	Materials and methods
recombinant DNA reagent	pF(UG) hSyn SYP-GCaMP6f	(Bradberry and Chapman, 2022)	Addgene	Materials and methods
recombinant DNA reagent	pCAG mito-GCaMP5G	Addgene	Addgene plasmid # 105009 ; http://n2t.net/addgene:105009 ; RRID:Addgene_105009	
Antibody	anti-MFN2 (mouse monoclonal)	Abcam; Abnova	Abcam Cat# ab56889, RRID:AB_2142629; Abnova Cat# H00009927-M01, RRID:AB_714775	IB (1:1000)
Antibody	anti-SYT1 (mouse monoclonal)	DSHB	mAB 48; RRID:AB_2199314	IB (1:1000) ICC (1:100)
Antibody	anti-SYP (guinea pig polyclonal)	Synaptic Systems	101 004; RRID:AB_1210382	IB (1:1000) ICC (1:500)
Antibody	anti-VDAC (rabbit polyclonal)	EMD/ Sigma-Aldrich	AB10527|Anti-VDAC Antibody; RRID:AB_10806766	IB (1:1000)
Antibody	anti-LAMP1 (rabbit polyclonal)	Abcam	ab24170; RRID:AB_775978	IB (1:500)
Antibody	anti-PSD95 (mouse monoclonal)	Thermo Fisher Scientific	Clone 7E3-1B8; catalog # MA1-046; RRID:AB_2092361	IB (1:1000)
Antibody	anti-SYB2 (mouse monoclonal)	Synaptic Systems	Clone 69.1; catalog #104 211C5, RRID:AB_2619757	IB (1:1000)
Antibody	anti-GYR1 (rabbit polyclonal)	Synaptic Systems	103 002, RRID:AB_887818	IB (1:1000)
Antibody	goat anti-guinea pig IgG-HRP	Abcam	ab6908, RRID:AB_955425	IB (1:10,000)
Antibody	goat anti-rabbit IgG-HRP	Bio-Rad	1706515, RRID:AB_11125142	IB (1:10,000)
Antibody	goat anti-mouse IgG2b-HRP	Bio-Rad	M32407; RRID:AB_2536647	IB (1:10,000)
Antibody	goat anti-mouse IgG-HRP	Bio-Rad	1706516; RRID:AB_11125547	IB (1:10,000)
Chemical	MitoTracker Green FM	Thermo Fisher Scientific	M7514	
Chemical	MitoTracker Red CMXRos	Thermo Fisher Scientific	M7512	
Chemical	Folimycin / Concanamycin A	Tocris Bioscience	2656	
Chemical	Hydrogen peroxide	Sigma-Aldrich	95321-500ML	Materials and methods
Chemical	Dithiothreitol	GoldBio	DTT100	Materials and methods
Chemical	Antimycin A	Sigma-Aldrich	A8674-25MG	Materials and methods
Chemical	Rotenone	MedChemExpress	HY-B1756	Materials and methods
Chemical	Trichloroethanol (TCE)	Sigma	T54801-100G	Total protein stain
Material	XONA microfluidic	XONA	SND450	Standard neuron microfluidic
Chemical	HTL-JF646-BAPTA-AM	Janelia Research Campus/HHMI	A kind gift from Dr. Luke Lavis	Luke Lavis Lab (Deo et al., 2019)

## Significance statement

Decreased or altered mitochondrial function is observed in many disease states. All neurodegenerative diseases co-present with some sort of mitochondrial abnormality. Therefore, identifying quality control mechanisms that sustain the mitochondrial network in neurons, and particularly in axons, is of significant interest. The response of axonal mitochondria to acutely applied toxins or injury has been studied in detail. Although informative, the response of neurons to these insults might not be physiologically relevant, so it is crucial to also study the basal behavior of axonal mitochondria. Here, we use fluorescent biosensors to investigate the mitochondrial network in neurons and examine the role of mitofusin 2 in maintaining the axonal mitochondrial network and in supporting the synaptic vesicle cycle.
